# Mechanisms governing codon usage bias and the implications for protein expression in the chloroplast of *Chlamydomonas reinhardtii*

**DOI:** 10.1111/tpj.15970

**Published:** 2022-10-19

**Authors:** Maxime Fages‐Lartaud, Kristoffer Hundvin, Martin Frank Hohmann‐Marriott

**Affiliations:** ^1^ Department of Biotechnology Norwegian University of Science and Technology Trondheim N‐7491 Norway; ^2^ United Scientists CORE (Limited), 2 Tewsley St Dunedin 9016 New Zealand

**Keywords:** chloroplast, genetic code, codons, codon usage bias, tRNA anticodon, protein expression

## Abstract

Chloroplasts possess a considerably reduced genome that is decoded via an almost minimal set of tRNAs. These features make an excellent platform for gaining insights into fundamental mechanisms that govern protein expression. Here, we present a comprehensive and revised perspective of the mechanisms that drive codon selection in the chloroplast of *Chlamydomonas reinhardtii* and the functional consequences for protein expression. In order to extract this information, we applied several codon usage descriptors to genes with different expression levels. We show that highly expressed genes strongly favor translationally optimal codons, while genes with lower functional importance are rather affected by directional mutational bias. We demonstrate that codon optimality can be deduced from codon–anticodon pairing affinity and, for a small number of amino acids (leucine, arginine, serine, and isoleucine), tRNA concentrations. Finally, we review, analyze, and expand on the impact of codon usage on protein yield, secondary structures of mRNA, translation initiation and termination, and amino acid composition of proteins, as well as cotranslational protein folding. The comprehensive analysis of codon choice provides crucial insights into heterologous gene expression in the chloroplast of *C. reinhardtii*, which may also be applicable to other chloroplast‐containing organisms and bacteria.

## Significance Statement

This work provides new insights into codon usage bias in chloroplasts. It is crucial for codon optimization of heterologous genes and to understand gene regulation in chloroplasts.

## INTRODUCTION

Codons are the fundamental link between genes and proteins. This position makes codons the target of a complex array of evolutionary forces. By analyzing the interactions of these forces, we may uncover fundamental connections and learn how to apply them in biotechnological applications.

**Figure 1:**
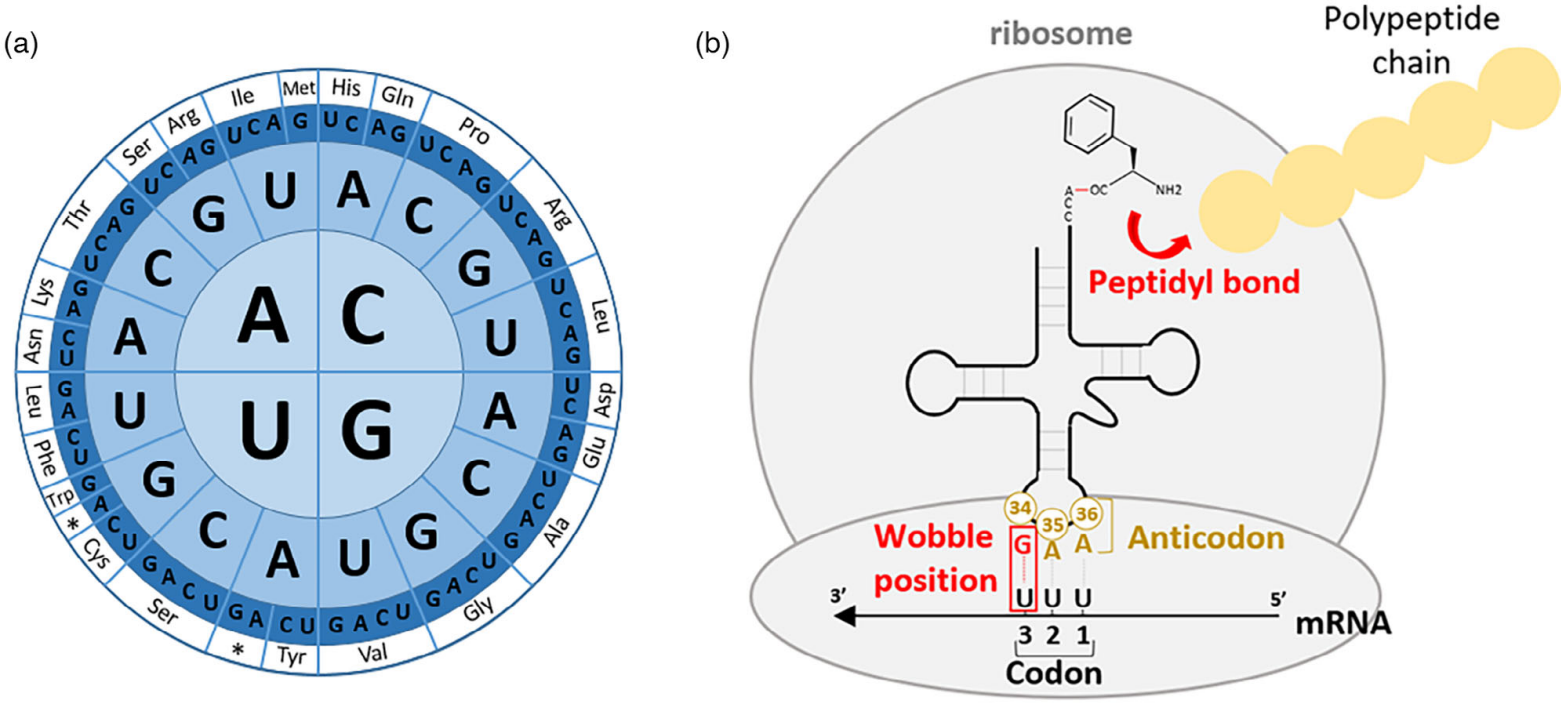
Overview of the genetic code and tRNA–mRNA interactions during translation. (a) Graphic representation of the genetic code. Codons are read from the letter in the center outwards. Corresponding amino acids are indicated by the three‐letter code and an asterisk for stop codons. (b) Pairing of a codon with the anticodon of an aminoacyl‐tRNA in the ribosome P‐site before peptide elongation.

Genetic information is universally encoded in DNA and RNA through sequences of nucleotides (A, T/U, C, and G). The genetic information encoding proteins is organized into nucleotide triplets called codons, offering 4^3^ = 64 possible combinations for encryption. To translate DNA into proteins, 61 triplets code are used for the 20 canonical amino acids and three are used to terminate translation (UAA, UAG, and UGA). The excess of possible nucleotide triplet combinations versus the number of codable amino acids leads to redundancy of the genetic code, where one amino acid is encoded by several codons (see Figure 1a). The segregation of the genetic code into codon families or ‘boxes’ is a consequence of nucleotide base pairing rules. Codon decryption is achieved through specific pairing with the anticodon of a tRNA that carries a specific amino acid. The codon bases at positions 1, 2, and 3 pair with positions N_36_, N_35_, and N_34_ of the anticodon loop, respectively (anticodon positions 3, 2, and 1, respectively) (see Figure 1b). Codon–anticodon recognition follows Watson–Crick pairing rules (A:U, U:A, G:C, C:G) for the first and second positions of the codon with bases N_36_ and N_35_ of the anticodon. In contrast, the interaction between the third codon position and the first anticodon base (N_34_) is less specific and follows an extended set of combinations expressed in the ‘wobble rules’ (Agris, 2004; 1991; Crick, 1966). In addition, a plethora of nucleotide modifications in the anticodon loop, especially in the wobble position N_34_ and anticodon adjacent N_37_, modulate codon discrimination (Agris, 2008; Osawa et al., 1992) by increasing or decreasing codon–anticodon pairing specificity. Therefore, with the exception of methionine and tryptophan, amino acids are often associated with several near‐cognate codons in duet, triplet, or quartet boxes, or even possess two decoding boxes (e.g., serine, leucine, and arginine) (see Figure 1a).

The precision of the genetic code relies on the supply of correctly charged aminoacyl‐tRNAs. Amino acids are ligated onto their corresponding tRNAs by specific aminoacyl‐tRNA synthetases. This specificity constitutes a crucial step in preserving the fidelity of the genetic code. Mature aminoacyl‐tRNAs enter the ribosomal A‐site and pair with anticodons by complementarity rules. Accurate codon–anticodon pairing allows movement into the ribosome P‐site. Here, a peptidyl bond is created with the previous amino acid of the nascent polypeptide chain (see Figure 1b). The tRNA exits the ribosome and the process is iterated to create mature proteins.

Historically, synonymous codons were considered equivalent because they resulted in ‘silent’ mutations with no consequences on the protein sequence. However, although the genetic code is nearly universal, it became evident that its utilization varies across species depending on the deciphering optimization strategy imposed by evolutionary forces (Grosjean et al., 2010; Grosjean & Westhof, 2016). In consequence, codon usage profiles differ widely across species. Furthermore, codon usage differs within the same species and correlates with gene expression in unicellular organisms (Bennetzen & Hall, 1982; Gouy & Gautier, 1982; Lloyd & Sharp, 1992; Sharp & Cowe, 1991; Sharp & Li, 1986). The species preference for certain redundant codons is known as codon usage bias. Prokaryotes and eukaryotes developed increasingly complex regulatory mechanisms of their genetic codes that are reflected in codon usage bias. Codon usage bias is a consequence of a multi‐layered complexity combining genetic code deciphering properties with elaborated regulatory mechanisms that span the entire protein expression process. The deconvolution of these interdependencies is not trivial and renders the implications of codon usage for protein expression difficult to understand in their entirety.

Microorganisms with a simple genetic makeup can be useful targets for gaining insights in the complexity of the genetic code. *Mycoplasma*, Archaea, and organelles possess simple versions of the genetic code (Grosjean et al., 2010; Grosjean & Westhof, 2016; Osawa et al., 1990, 1992) and are targets for exploring the mechanisms underlying codon usage bias. For example, *Mycoplasma capricolum* and mitochondria use UGA as a tryptophan codon instead of a stop codon (Grosjean & Westhof, 2016); mitochondria leave the arginine duet‐box AGA/G unassigned (Grosjean & Westhof, 2016); while Archaea exhibit a reassignment of the UAG stop codon to the non‐canonical amino acid pyrrolysine (Ambrogelly et al., 2007). These deviations from the ‘universal’ code can be viewed as relics of an ancestral genetic code that is more than a billion years old (Jukes, 1973) or as the result of an evolutionary sparing strategy imposed by small tRNA sets (Grosjean et al., 2010; Grosjean & Westhof, 2016). The ancestral genetic code proposed by Jukes in 1973 (Jukes, 1973) coded for 10 amino acids before expanding to the contemporary code due to increasing encrypting capacities introduced by tRNA modifications. Organelles, such as mitochondria and chloroplasts, present a minimal tRNA set with a reduced complexity in tRNA modifications. This apparent simplicity makes the deciphering of their genetic code highly dependent on physical properties of nucleotides. Thus, organelles like chloroplasts are entities of choice to study the first layers of complexity of the genetic code and its evolution.

In this study, we focus on the chloroplast of the model organism *Chlamydomonas reinhardtii*, although this work may be applicable to other chloroplast‐containing organisms due to a common evolution (Suzuki & Morton, 2016). Plant and microalgal plastids represent attractive platforms for biotechnological applications, including the production of energy, therapeutics, animal food, and high‐value nutritional and biochemical coproducts for the industry (Almaraz‐Delgado et al., 2014; Bock, 2015; Cardi et al., 2010; Doron et al., 2016; Dyo & Purton, 2018; Rosales‐Mendoza et al., 2012; Scaife et al., 2015; Scranton et al., 2015; Specht et al., 2010). Biotechnological applications and fundamental research can take advantage of a sophisticated set of genetic tools that have been developed for the chloroplast (Bock, 2015; Doron et al., 2016; Scaife et al., 2015; Wang et al., 2009). Expressing genes is an essential outcome for most biotechnological work in chloroplasts. It is therefore important to understand each aspect of gene expression to realize the biotechnological potential of the chloroplast. The utilization of codon optimization tools in chloroplasts with methodologies established in bacteria (Weiner et al., 2020) ignored some key aspects of codon usage bias and its impact on gene expression that we present in this study.

The evolutionary history of the chloroplast is reflected by the organization of its protein expression machinery. Chloroplasts originate from endocytosis, i.e., the engulfment of an ancient member of the cyanobacterial clade (Douglas & Turner, 1991; Gray, 1989; Martin & Kowallik, 1999). The ensuing symbiotic relationship gave rise to metabolite exchange, deletion of dispensable genes or transfer of essential genes from the plastome to the nucleus, and the subsequent development of a protein import machinery that reroutes nuclear gene products to the chloroplast (Bock, 2015; Scharff & Bock, 2014). Consequently, the polyploid plastome was considerably reduced so its size slightly exceeds a couple hundred kilobases and contains on average 120 genes (Bock, 2015; Dyo & Purton, 2018; Gallaher et al., 2018). The majority of plastid genes are involved in photosynthesis and in the chloroplast's personal transcription/translation apparatus and ATP synthesis (Gallaher et al., 2018; Maul et al., 2002). This small prokaryotic‐like genome is maintained probably due to the necessity of protein coexpression with either cofactors or nuclear‐encoded counterparts (Stern et al., 2010).

Transcription in the plastid is mediated by two types of RNA polymerases, the nuclear‐encoded T7 phage‐type polymerases (NEPs) and the eubacterial plastid‐encoded polymerases (PEPs) (Hess & Börner, 1999; Shiina et al., 2005). Chloroplastic gene expression is regulated by various nuclear‐encoded sigma factors, which activate translation of proteins involved in abiotic stress responses, light and redox signals, and development, depending on promoter type (Barkan, 2011; Kanamaru & Tanaka, 2004). After transcription, mRNAs are processed. Polycistronic transcripts are cleaved into smaller fragments and stabilized by sequence‐specific tetra‐, penta‐, or octa‐tricopeptide repeat (TPR, PPR, and OPR) proteins (Barkan, 2011; Del Campo, 2009; Jalal et al., 2015; Raynaud et al., 2007; Schmitz‐Linneweber & Small, 2008; Shikanai & Fujii, 2013; Stern et al., 2010). The chloroplast translation machinery, composed of prokaryotic orthologs, proceeds to translation of mRNA into proteins.

Plastids encode their own almost minimal set of tRNAs, which is often close to the minimal 25‐tRNA set required to decipher the genetic code (Alkatib et al., 2012), and there is no evidence of tRNA import from the nucleus (Duchene et al., 2005; Marechal‐Drouard et al., 1993). In the chloroplast of *C. reinhardtii*, regulation of protein expression occurs mainly at the translation level (Veronica et al., 2004) by *cis*‐elements such as the Shine–Dalgarno (SD) sequence (Scharff et al., 2017; Shine & Dalgarno, 1974; Weiner et al., 2019), mRNA secondary structure (Mauger et al., 2013; Scharff et al., 2011), codon usage (Nakamura & Sugiura, 2007; Pfitzinger et al., 1987), non‐coding RNA (Anand & Pandi, 2021; Dietrich et al., 2015), nascent peptide elements (Zoschke & Bock, 2018), and *trans*‐elements like the abovementioned PPR proteins.

In this paper, we investigate the effects of codon usage along the entire protein expression process. We enrich our understanding of the functional aspects by including key subtleties to obtain a global and accurate view of codon usage regulation in chloroplasts. We provide an overview of molecular mechanisms that underlie codon usage bias and explore the implications on protein expression. Our work advances the interpretations of codon usage bias in the chloroplast beyond the boundaries of existing literature.

## RESULTS AND DISCUSSION

### Examination of the codon usage bias fingerprint of the chloroplast

In the chloroplast, codon usage is traditionally analyzed for the total coding sequences (CDSs) of the genome. However, previous studies showed that codon usage can vary widely between functional sets of genes within a single organism (Osawa et al., 1990, 1992). This complexity has often been overlooked or was not investigated in its entirety in the chloroplast. In this section, we will develop and apply codon usage descriptors to define and assess the codon usage bias of the chloroplast in more detail.

#### Codon usage bias correlates with gene expression

The presence of an intraspecies codon usage bias between genes with different expression levels was observed for a wide range of organisms (Bennetzen & Hall, 1982; Gouy & Gautier, 1982; Lloyd & Sharp, 1992; Sharp & Cowe, 1991; Sharp & Li, 1986). This bias can be estimated by the Codon Adaptation Index (CAI) (Sharp & Li, 1987), in which the codon usage of each gene is compared to the supposed codon optimality of a reference set composed of highly expressed genes (see Methods). Codon usage bias profiles were correlated with tRNA gene copy number, or more accurately tRNA concentrations (Duret, 2000; Ikemura, 1982; 1981), and can be assessed with the tRNA Adaptation Index (tAI) (dos Reis et al., 2004). In the chloroplast, the tRNA gene copy number is either one or two, rendering this correlation inadequate (Data S3). The tRNA reads from RNA sequencing (RNAseq) data (Gallaher et al., 2018) do not accurately represent the mature tRNA population because of the difficulty in detecting such structurally complex RNAs and it ignores aminoacyl‐tRNA maturation. Interestingly, quantification from 2D PAGE migration showed a high correlation between amino acid occurrence and tRNA concentrations (Pfitzinger et al., 1987). Although this analysis is valid for leucine, serine, and arginine, all possessing two distinct codon boxes each, and for the special case of isoleucine's three‐codon box, it is not suitable to explain codon bias for other amino acids since only one tRNA is responsible for reading all their corresponding codons. Therefore, we used CAI to investigate the presence of a codon usage bias in the chloroplast associated with gene expression.

**Figure 2:**
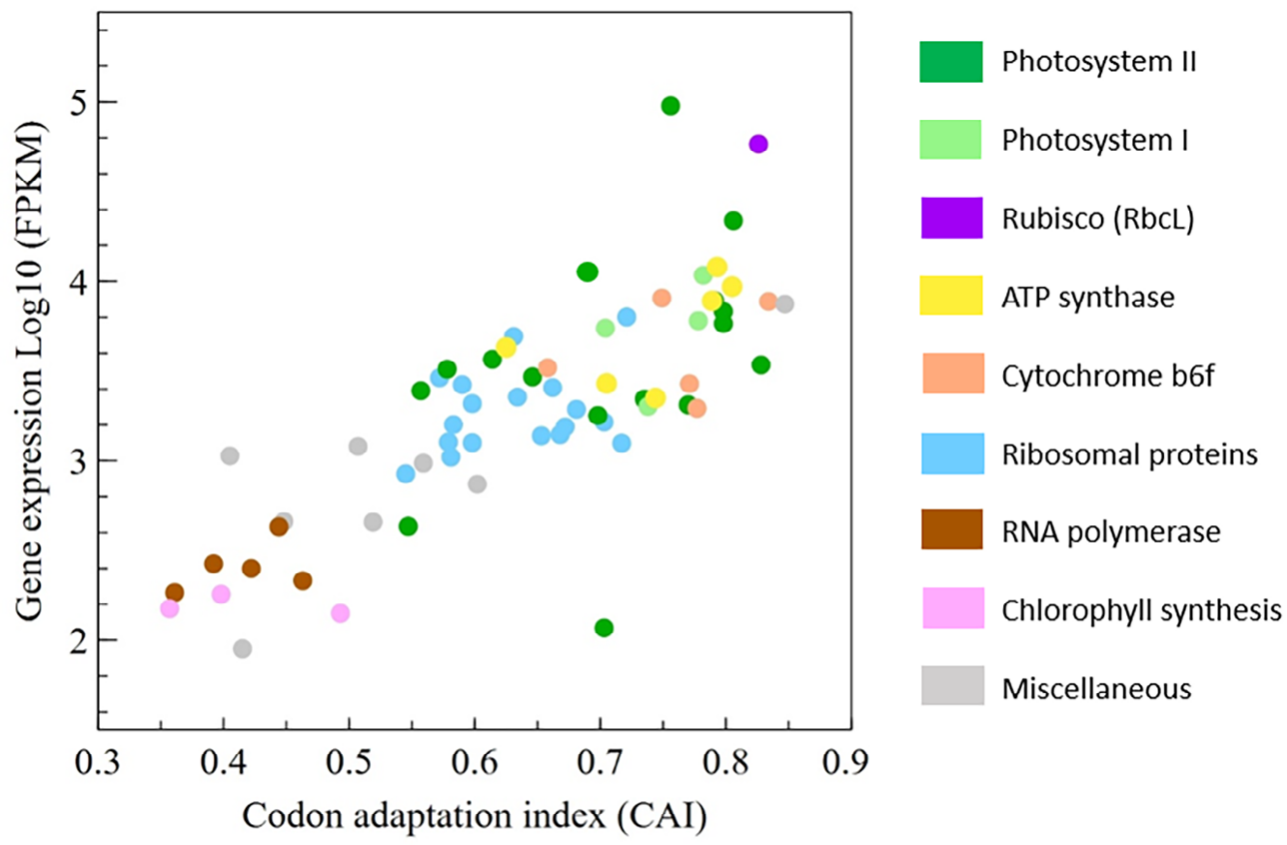
Correlation between gene expression (mRNA FPKM values) and the Codon Adaptation Index (CAI) for CDSs of the *C. reinhardtii* chloroplast (Pearson coefficient 0.78). Categories of functional genes are represented in different colors. Reference set = Top 18 (see Methods).

The CAI of each gene was calculated and plotted against its mRNA expression level (fragments per kilobase of exon per million mapped fragments [FPKM]). Transcript levels were used as a proxy for gene expression, as it was previously shown that transcript levels and protein content often correlate in their native cellular environment (Bennetzen & Hall, 1982; Gouy & Gautier, 1982; Ikemura, 1981; Pfitzinger et al., 1987), despite variations in translation efficiency. A strong correlation (Pearson coefficient = 0.78) was identified between codon adaptation and gene expression (Figure 2). This correlation demonstrates the strong preference for certain codons in highly expressed genes (Top 18 genes). Interestingly, functional groups of genes are distributed according to this correlation. Genes responsible for photosynthesis, such as genes encoding components of photosystems I and II, Rubisco (*rbcL*), cytochrome *b*_*6*_ *f*, and ATP synthase, display both high CAI and high expression. A certain correlation is expected by design, since CAI is based on a reference set that consists of highly expressed genes. However, the aforementioned genes also cluster together when a smaller reference set is used (Top 7 genes), demonstrating the weak effect of the choice of reference group. Only *psbI* is far outside the correlation; this may be explained by a systematic RNAseq under quantification due to particular mRNA instability or secondary structure, or high translation efficiency, protein stability, and protein turnover, or may be related to the function of *PsbI* (Wang et al., 2015). In contrast, genes responsible for chlorophyll synthesis and RNA polymerase (PEP) subunits are the two functional groups with low expression and a significantly different CAI (Low 8 group). The pool of ribosomal proteins is intermediate and appears just slightly biased.

We also performed an analysis of codon usage bias within previously identified operons (Shahar et al., 2019), in an attempt to identify a correlation between codon usage bias and operon expression (Data S1). Some operons show good CAI versus mRNA level correlation (e.g., *rpl2‐rps19*, *rpl16‐rps14*, *rps18‐ycf3*, and *psbJ‐atpI‐psaJ‐rps12*). However, other operons, despite showing similar CAI values, possessed different transcript levels for their respective genes (e.g., *psaC‐petL*, *psbT‐psbB*, *rps8‐psaA_exon1*, *psaA_exon2‐psbD*). This suggests the presence of gene‐specific promoters within each operon, decoupling the quantitative transcript levels of each gene despite a basal readthrough for the complete operon.

#### Analysis of codon optimality in the chloroplast

The notion of codon optimality refers to translation efficiency and is different from total codon frequency at the genomic level. This distinction has often been disregarded and led to ambiguous interpretations of the genetic code optimality in the chloroplast (Alkatib et al., 2012; Nakamura & Sugiura, 2007). Since biased, highly expressed genes can constitute a relatively small group in comparison to the total number of CDSs of a cell, the codon optimality information they contain is diluted among the evolutionary constraints of the majority. Ideally, codon demand should consider protein levels quantitatively and include cellular dynamics such as protein stability and turnover, mature aminoacyl‐tRNA concentrations, and tRNA modifications. For example, a cell regulates tRNA aminoacylation and tRNA modifications to express different genes under a range of conditions (Jayabaskaran et al., 1990). However, obtaining comprehensive data to analyze this is tremendously demanding. A common simplification is to ignore translation regulation processes and use transcriptomic data (mRNA and tRNA) or genomic data while including a gene expression component (such as CAI) (Bennetzen & Hall, 1982; Gouy & Gautier, 1982; Ikemura, 1981; Pfitzinger et al., 1987). In order to extract this information from the chloroplast, we analyzed the number of each codon, their frequency per 1000 codons, the Relative Synonymous Codon Usage (RSCU), and the relative adaptiveness of a codon (W_ij_) (see Methods for calculations) (Data S2). This analysis was performed on total CDSs, as well as the high‐ and low‐expression groups (Top 18 and Low 8, respectively). Using the high‐expression group, these calculations permit to identify ‘optimal’ and ‘non‐optimal’ codons as occurring frequently and rarely, respectively. The fold differences between groups were also calculated (Data S2).

**Figure 3:**
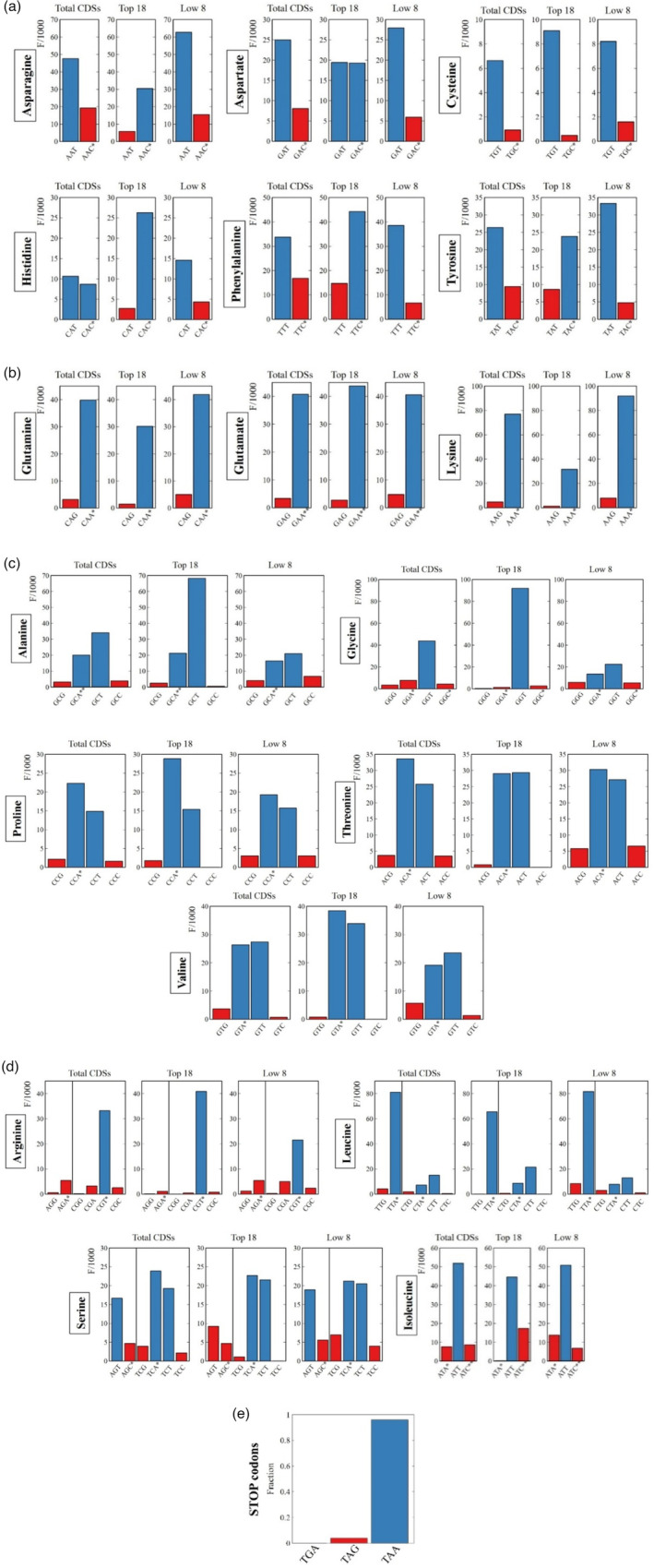
Codon usage frequency statistics. Histograms represent the frequency per thousand of each codon in DNA CDSs. The analysis was made for total CDSs and expression groups Top 18 and Low 8. Blue shows the preferential codon(s) compared to the less preferred codon in red for each group. Asterisks mark the presence and the copy number of corresponding tRNAs. (a) The NNU/C duet boxes show an enrichment in NNC codons in high‐expression genes while NNU is preferred in low‐expression genes. The total codon sequences hide the optimality of codons of highly expressed genes. (b) For the NNA/G duet boxes, NNA is always favored across all expression groups and NNG is repressed in high‐expression genes. (c, d) For quartet boxes, NNA/U codons are always favored (glycine and arginine favor only their respective NNU codon), while NNG/C codons are also repressed in high‐expression genes. For amino acids with several tRNAs, we can identify the favored tRNA isoacceptor: the quartet boxes of serine and arginine, the duet box of leucine and isoleucine. (e) Stop codons are almost exclusively TAA; three TAG codons and no TGA codons are present.

**Figure 4:**
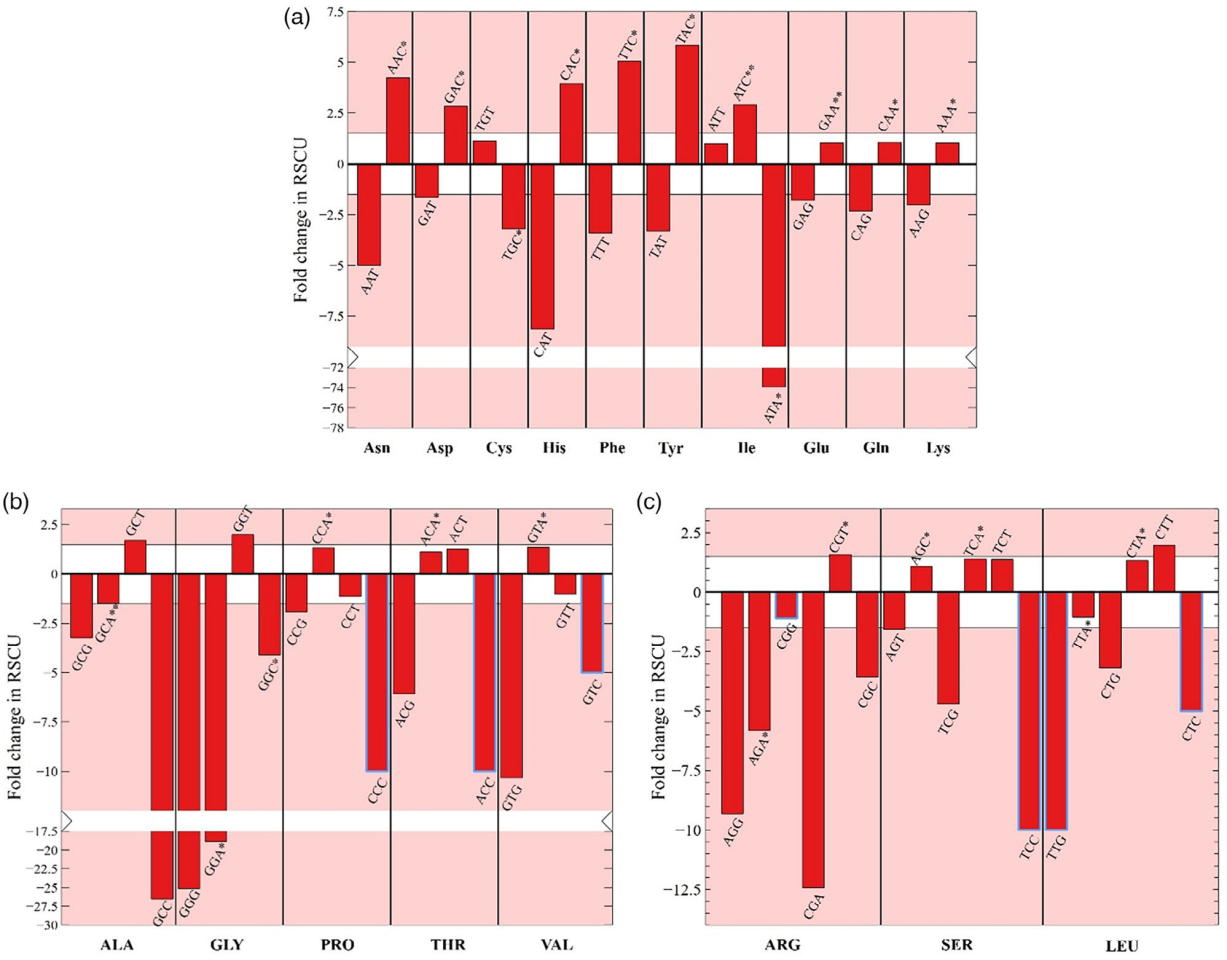
RSCU fold difference. Differences in RSCU for each codon between Top 18 and Low 8 expression groups for (a) duet boxes, (b) quartet boxes, and (c) six‐codon boxes are displayed. Asterisks mark the presence of a cognate tRNA and its copy number. The red zones delineate >1.5‐fold change in RSCU. Blue rectangles show artificially set fold change values because of the complete absence of the codon in the Top 18 group.

**Figure 5:**
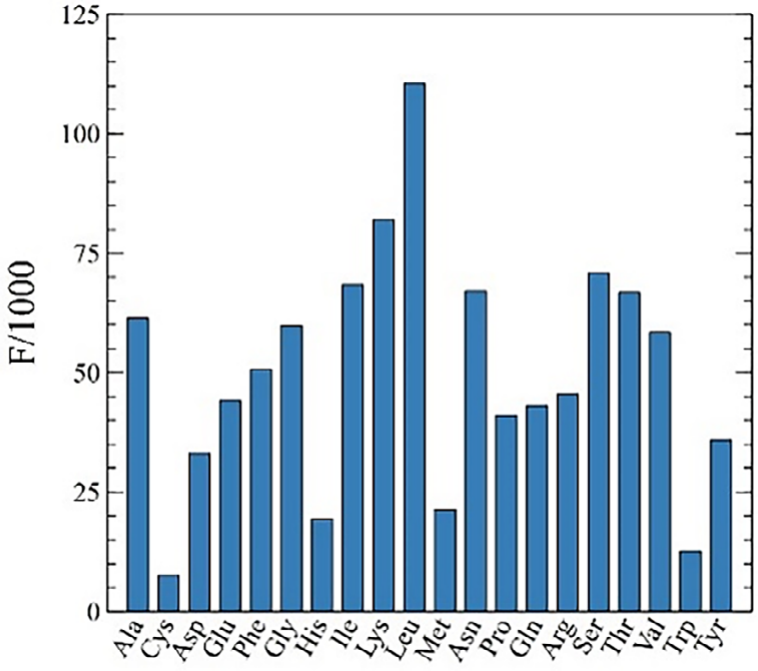
Genome‐wide codon frequency. Frequency per thousand of amino acid usage in *C. reinhardtii*'s chloroplast CDSs.

The results of the frequency per thousand calculations are presented in Figure 3 and permit to distinguish three categories of codons. The first group is composed of the NNU/C duet boxes (Asn, Asp, Cys, His, Phe, and Tyr). The low‐expression group is richer in NNU codons, while high‐expression genes show significant opposite enrichment with NNC codons (Figures 3a and 4). Only cysteine is an exception to this rule; in all expression groups UGU is preferred. Cysteine may be a special case due to its low occurrence in proteins (Figure 5), a particular tRNA architecture, or its involvement in protein tertiary structure through the formation of disulfide bridges. Isoleucine AUU/C and serine AGU/C can be included in this group (Figure 3d). Even though the isoleucine codon AUC appears more often in high‐expression genes, AUU remains the favored codon for this amino acid across all expression groups. However, its third synonymous codon (AUA) is almost absent from the Top 18 group and relatively common in the Low 8 group, suggesting that another regulatory mechanism is driving codon choice for isoleucine. For the duet box of serine, the AGU codon is less used in high‐expression genes, but it is compensated by using favored codons of the quartet box (UCA/U) rather than the AGC codon. The RSCU fold differences for the NNU/C duet boxes suggest that C‐ending codons are favored in high‐expression genes, while U‐ending codons are overrepresented in the low‐expression group (Figure 4).

A second group of codons can be identified by the possession of NNA/G duet boxes (Glu, Gln, Lys, Leu [UUA/G], and Arg [AGA/G]). In this group, the A‐ending codon is always favored and there is a slight increase in G‐ending codons in low‐expression genes (Figures 3b and 4). The duet box of arginine (AGA/G) follows the same rule, although its usage is relatively low in comparison to the quartet box (CGN) and almost absent in the Top 18 expression group. The NNA/G duet boxes show less variation across expression groups, suggesting a lower impact on codon regulation compared to the NNU/C duet boxes.

Finally, the group of quartet boxes (Pro, Arg, Leu, Ala, Gly, Val, Ser, and Thr) shows a strong preference for A/U‐ending codons regardless of gene expression levels. Nevertheless, G/C‐ending codons are more frequent in the low‐expression group (Figures 3c,d and 4). This G/C‐ending codon bias could have a potential influence on codon regulation even though it might be moderate considering the low occurrence of these codons. Exceptions to these rules are glycine and arginine, which both strongly favor only their respective U‐ending codons in the quartet box.

Using a detail‐rich analysis our study reveals a codon optimality that deviates from studies that use bulk genome‐wide codon usage to determine ‘optimality’. Our study suggests that codon optimality is hidden in a relatively small set of highly expressed genes and becomes obscured by including unweighted genome‐wide codon usage. The main overlooked codon optimality concerns the group of NNU/C duet boxes that display opposite preference between highly expressed genes and genome‐wide codon usage.

In order to refine codon representation in transcripts and aminoacyl‐tRNA demand during translation, we estimated the representation of each codon in mRNA by combining sequences with expression data (Data S1). Indeed, even though highly expressed genes are adapted to higher tRNA concentrations (Duret, 2000; Ikemura, 1982; 1981), the codon overrepresentation in highly expressed mRNAs will consume the respective tRNAs at a higher rate, and this is likely to even out the tRNA concentration effects. Since the chloroplast mainly contains one tRNA per codon box, this analysis should support the frequency per thousand and CAI results at the genomic level. As expected, codon demand reinforces the distinction into three codon groups found with genomic frequency per thousand (Data S2). This analysis also provides a finer‐grained perception of the preponderance of the bias between expression groups (Data S1). The average bias between Top and Low expression groups for NNU/C duet boxes, NNA/G duet boxes, and quartet boxes is 20.9‐, 3.1‐, and 13.3‐fold, respectively, displaying their effect on translation regulation. We constructed a list of optimal and non‐optimal codons and a codon usage table of the Top expression group that can be used for codon optimization of heterologous genes (Data S1).

**Figure 6:**
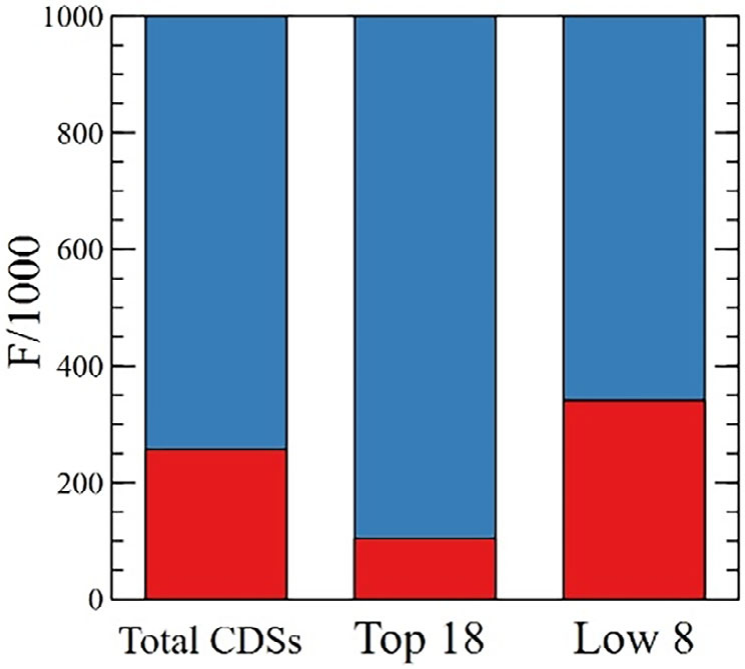
Rare and frequent codons. Frequency per thousand of all rare codons (red) and high‐frequency codons (blue) for total CDSs, the Top 18 group, and the Low 8 group.

Overall, optimal codons represent 89.6% of codons in high‐expression genes, 74.3% in total CDSs, and only 65.9% in the low‐expression group (Figure 6). As demonstrated in this section, codons can be arranged into three groups that have different weights in codon bias. The natural question that ensues concerns the origin of this bias, as well as its function and its biological implications.

### Mechanisms responsible for codon usage bias in the chloroplast

In this section, we will explore forces that shape the nucleotide and the codon composition of the plastome. First, we describe how the evolutionary mutational bias directed the nucleotide composition to reach the genomic signature of the chloroplast. Then, we investigate the forces that influenced the codon composition of CDSs to deviate from the genomic nucleotide signature.

Based on studies in *Escherichia coli* and yeast, Ikemura proposed two evolutionary forces dictating codon selection. He formulated the following rules: (i) when two different tRNA isoacceptors are present for the same amino acid, the codon with the most abundant tRNA is used more frequently; (ii) when only one tRNA reads several codons through wobble base pairing, a higher affinity between codon and anticodon leads to a higher usage (Ikemura, 1982, 1981). We examined the preponderance of both rules for shaping the codon usage of the chloroplast. Finally, we examine potential mechanisms thought to influence the choice of codon juxtaposition.

#### Evolutionary mutational bias shapes nucleotide composition

The genomic nucleotide composition varies widely across bacterial species from 25 to 75% GC content (Osawa et al., 1990). These variations are the consequence of directional substitution mutation rates (toward A:T or C:G) and the mutational equilibrium represents the genomic signature of an organism (Sueoka, 1988; 1962). Mutation rates are intrinsically associated with the DNA replication and repair systems. On the one hand, replication may generate context‐dependent mutations or operate more efficiently on specific sequences (Karlin et al., 1997). On the other hand, variations in composition of DNA repair enzymes, such as the *mut* gene family, can promote transversions from A:T to C:G or inversely (Bai & Lu, 2007; Cabrera et al., 1988; Denamur et al., 2000; Fowler & Schaaper, 1997; Nghiem et al., 1988). This resulting organism‐specific GC content is a result of selective constraints exerted to eliminate deleterious mutants and optimize cell growth (Kimura, 1991; Osawa et al., 1992). In opposition to Darwinian evolution processes, Kimura formulated the neutral theory of evolution, stating that genetic regions with lower functional importance evolve through random nucleotide fixation (Kimura, 1991). The neutral sites in question are constituted of genetic spacer regions and protein and DNA polymorphisms (i.e., amino acid similarities [Grantham, 1974; Yampolsky & Stoltzfus, 2005] and third codon positions). This phenomenon is referred to as evolutionary directional mutational bias (Bulmer, 1990; Sharp & Li, 1986). An asymmetric, strand‐specific mutational bias has also been described (Lobry & Sueoka, 2002).

It was demonstrated that the GC content of all three codon positions presents a positive linear correlation with genomic GC content (Muto & Osawa, 1987). Under the neutral theory of evolution assumption, the GC content in the third codon position (GC3) should match the expected value from the genomic correlation. The genomic GC content of the chloroplast is 34.5 and 31.9% for CDSs and 36.1% for non‐CDS DNA. The expression groups Top 18 and Low 8 present GC contents of 39.0 and 30.9%, respectively, which, based on the neutral theory of evolution, should result in GC3 values close to 34 and 19%, respectively. The GC content was analyzed for all three codon positions across the expression groups (Data S2). The GC3 value of the low‐expression group is 16.2 ± 2.5%, which is close to the expected value of 19%. Therefore, the codon usage of genes with lower functional importance follows the rules of the neutral theory of evolution and evolutionary mutational bias. However, the highly expressed gene set possesses a GC3 content of 20.6 ± 7.6%, which is much lower than the expected 34%.

Additionally, the GC3 content between expression groups deviates more drastically from the genomic correlation when considering the three groups of codons established in the previous section (Data S2). While the low‐expression group generally follows the neutral theory, it is noteworthy that the NNA/G duet box is slightly underrepresented in NNG codons (11.5 ± 4.5%). This indicates an active selection to decrease NNG codons that could be unfavorable for protein expression. In the case of highly expressed genes, the third codon position analysis draws the same conclusion regarding codon overrepresentation among codon family boxes. In brief, NNA/G duet boxes and quartet boxes show very low GC3 percentages (2.5 ± 1.0 and 5.4 ± 4.5%, respectively) strongly deviating from mutational bias predictions, while NNU/C duet boxes show a strong enrichment in NNC codons with the exception of cysteine (58 ± 31.7 and 66.8 ± 23.5% with and without including cysteine, respectively). The high deviation from the genomic GC content correlation for highly expressed genes indicates that there is another mechanism involved in codon selection than solely evolutionary mutational bias.

#### tRNA concentrations influence codon usage

In this section, we will explore the codon families that follow Ikemura's first rule: when two different tRNA isoacceptors are present for the same amino acid, the codon with the most abundant tRNA is used more frequently (Ikemura, 1981; Ikemura, 1982). In the chloroplast, only a few amino acids possess several tRNAs (Leu, Arg, Ser, Ile, and Gly). The isoleucine three‐codon box possesses a tRNA‐G_34_AU reading the two codons AUU and AUC; in addition, a second isoacceptor, tRNA‐k_2_C_34_AU, reads its third codon (AUA). As mentioned in Section 1, codon usage bias of the AUU/C duet box does not exactly follow the typical behavior of NNU/C duet boxes. Indeed, while there is an enrichment in AUC codons in high‐expression genes, the main codon for isoleucine remains AUU. Codon regulation seems to occur rather on the AUA codon, which is almost absent from the high‐expression group but common in the low‐expression group (Figures 3d and 4). We hypothesize that tRNA‐k_2_C_34_AU is rare in comparison to tRNA‐G_34_AU, thus making AUA the key regulatory codon for isoleucine. Through this mechanism, the effects of mutational bias, favoring A/T in the third codon position, do not have to be countered, while tRNA concentrations regulate isoleucine codon usage. For the four‐codon box of glycine, the combination of tRNA‐G_34_CC and tRNA‐U_34_CC reading properties favors the U‐ending codon but tRNA concentrations might not be a dominant factor of regulation. Moreover, tRNA‐G_34_CC has been shown to be dispensable in the chloroplast (Rogalski et al., 2008).

For each six‐codon box (Ser, Arg, and Leu), one tRNA reads a duet box and the other one reads a quartet box. For serine, the AGU codon from the duet box is less used in high‐ than in low‐expression genes, but it is compensated by the favored codons of the quartet box (UCA/U) rather than with the synonymous AGC codon of the duet box. Here, the usage of the duet box is lower but reasonable in high‐expression genes, while the duet box is used significantly in the low‐expression group. This suggests that the tRNA‐U_34_GA concentration is higher than that of the other isoacceptor. Additionally, the codon choice strategy for serine also lowers the GC3 content, in accordance with mutational bias pressure. In the case of arginine, the duet box AGA/G is the key of its codon regulation. It is rarely used in the high‐expression group but slightly more common in the low‐expression group. Thus, tRNA‐I_34_CG is preponderant compared to tRNA‐U_34_CU, and makes CGU the preferred codon. For the leucine six‐codon box, across the duet and quartet box, A/U‐ending codons are almost exclusively present in highly expressed genes, while C/G‐ending codons are more frequently found in low‐expression genes. The UUA codon is used most frequently, suggesting that the tRNA‐Um_34_AA concentration is higher than the tRNA‐U_34_AG concentration. However, it seems like C/G‐ending codons play a yet to be defined role in codon regulation for leucine.

The first rule of Ikemura helps to explain tendencies of codon usage for some codons but only for a few amino acids. Therefore, we will investigate in the following section how Ikemura's second rule affects the codon usage of the chloroplast.

#### tRNAs pairing properties are responsible for codon optimality within each codon family

Every organism possesses specific characteristics, such as a mutational bias signature, a certain tRNA set, and particular tRNA expression levels. Depending on these characteristics, evolution drove organisms to adopt different decoding strategies. These strategies have been categorized into four groups by Grosjean et al. (Grosjean et al., 2010). The chloroplast falls into the third sparing strategy, which consists in a total depletion of tRNA harboring A_34_ and C_34_ in the anticodon. Additionally, the ability of U_34_ to read all 4‐fold degenerate codons by superwobbling permits decryption of the genetic code with only 25 tRNAs (Alkatib et al. 2012; Rogalski et al., 2008).

**Figure 7:**
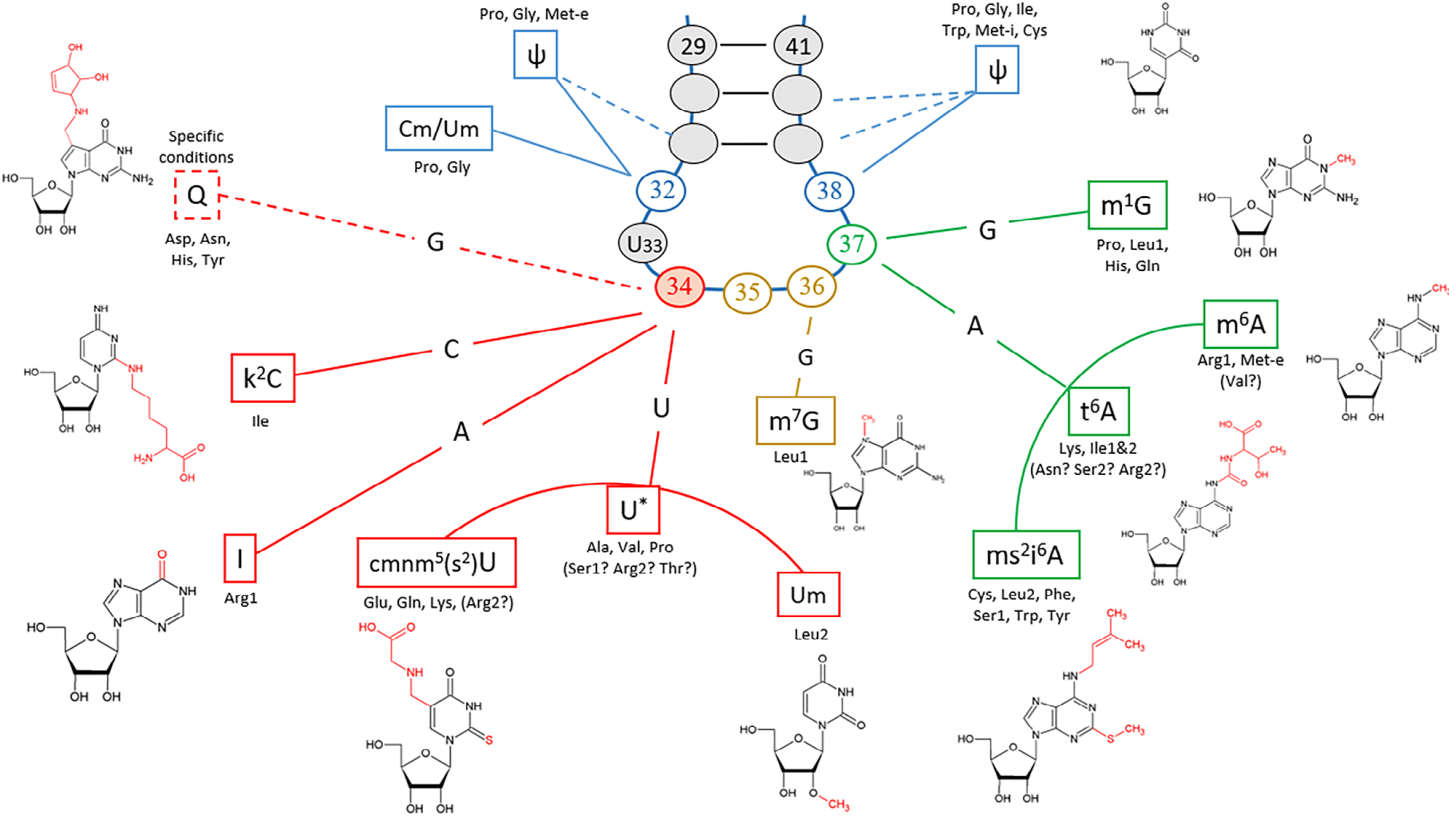
Principal modifications of the tRNA anticodon loop. The principal modifications of the tRNA anticodon loop are shown in connection to their position (Figure from Fages‐Lartaud & Hohmann‐Marriott, 2022, submitted). Each nucleotide position of the anticodon loop is associated with corresponding modifications depending on the type of original nucleotide. The modifications at position 37 (in green) maintain the decoding accuracy by avoiding interferences between codon boxes. The nature of anticodons, especially with the modifications of the wobble base N_34_ (in red), determines the codon–anticodon pairing affinity and defines codon optimality.

Ikemura's second rule states that when only one tRNA reads several codons by wobbling, a higher affinity between codon and anticodon leads to a higher usage (Ikemura, 1981, Ikemura, 1982). Optimization of the binding energy between codons and anticodons is a selection criterion that drives evolution. During translation, matching Watson–Crick base pairing provides an advantage over wobble base pairing by decreasing codon deciphering rates (Grosjean & Westhof, 2016; Letzring et al., 2010, Stadler & Fire, 2011). Importantly, Watson–Crick geometry and tRNA modifications are fundamental structural features that influence the acceptance of the appropriate tRNA species by the ribosome during translation, affecting its kinetics beyond purely codon–anticodon recognition (Cochella, 2005; Gromadski et al., 2006; Ogle et al., 2001, 2002). Pairing affinity is highly dependent on the identity of the anticodon, the respective nucleotide modifications, and tRNA secondary structures. In order to verify the applicability of Ikemura's second rule to codon selection in the chloroplast, we collated bioinformatics analysis with available experimental data to build a comprehensive picture of the current knowledge concerning tRNA modifications in the chloroplast (Figure 7) (Fages‐Lartaud & Hohmann‐Marriott, 2022). The results of this study are used to infer codon–anticodon affinity and translation efficiency.

**Figure 8:**
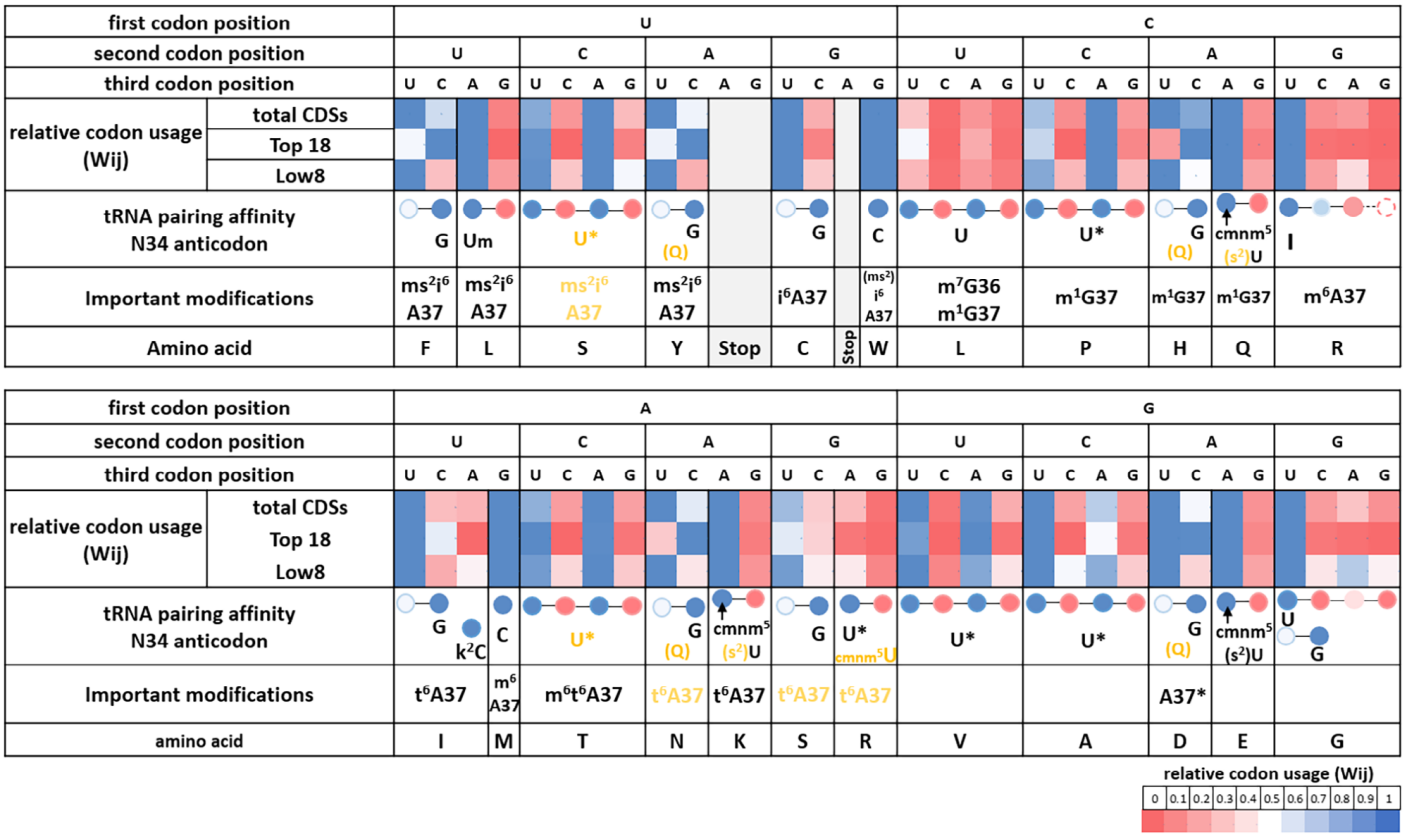
Correlation between tRNA properties and codon usage bias. Codon usage for total CDSs, and the high‐ and low‐expression groups (Top 18 and Low 8, respectively). Codon usage is displayed with tRNA modifications and their pairing affinities to show the effect of codon–anticodon pairing on codon usage bias. The relative codon usage (Wij) is represented for each codon box associated with an amino acid. The relative usage of the different codons encoding the same amino acid is indicated. More precisely, the subtlety of codon usage bias in highly expressed genes compared to the total CDSs or the low‐expression group (Wij is red for low toward blue for high) is displayed. The last row contains the tRNA modifications of anticodon position 34 and other important modifications. The codon–anticodon pairing efficiency is represented with the same color code as Wij (from Fages‐Lartaud & Hohmann‐Marriott, 2022). Base 34 of each anticodon is represented directly below the codon it recognizes by Watson–Crick pairing (whenever it is possible). Shown in black are modifications determined experimentally and shown in yellow are modifications postulated from bioinformatics analysis. This figure shows the correspondence between codon usage of highly expressed genes and codon–anticodon pairing efficiency. For amino acids with multiple tRNAs, it shows which isoacceptor is preponderant. Codon optimality is represented by the high‐expression group and correlates with codon–anticodon pairing affinity or tRNA concentration.

The chloroplastic tRNA set resembles the one of *M. capricolum* analyzed by Grosjean et al. in terms of tRNA modifications, the ability of U_34_ to read an entire quartet box, and a relatively similar GC content (Grosjean & Westhof, 2016). Substituents such as Um, cmnm^5^, and cmnm^5^s^2^ on U_34_ restrict anticodon pairing to NNA/G boxes with a strong preference for A‐ending codons (Fages‐Lartaud & Hohmann‐Marriott, 2022; Grosjean et al., 2010; Kurata et al., 2008; Lim, 1994; Takai & Yokoyama, 2003). Such a large difference in codon–anticodon affinity explains the omnipresence of NNA codons for duet boxes regardless of the expression groups (Figure 8). Additionally, favoring NNA codons is in accordance with the mutational bias; hence, there are no opposing evolutionary forces.

For the quartet boxes, the unmodified U_34_ nucleotide efficiently pairs with U‐ and A‐ending codons, while a G_3_:U_34_ wobble base is unstable and C‐ending codons are not read efficiently (Grosjean & Westhof, 2016). Anticodons containing an unknown modification of U_34_ were hypothesized to lead to higher translation efficiency of A‐ and U‐ending codons within quartet boxes in the chloroplast of *C. reinhardtii* and in *M. capricolum*, in accordance with Ikemura's second rule (Fages‐Lartaud & Hohmann‐Marriott, 2022; Grosjean & Westhof, 2016). These codon–anticodon affinities, plus the mutational bias, explain the strong codon usage bias toward NNU/A codons in quartet boxes across all gene expression groups (Figure 8). Once again, NNG/C codons are particularly repressed in high‐expression genes but not completely absent, which may be due to mutational bias or an underlying hidden functionality. The only exceptions to these rules are arginine and glycine quartet boxes. Indeed, the inosine of tRNA_Arg_‐I_34_CG pairs preferentially with U, slightly less with C, barely with A, and almost never with G. Therefore, arginine almost exclusively uses the CGU codon (Figure 8). For glycine, it was shown that its tRNA often favors exclusively the GGU codons for reasons that are not completely understood. A possible reason for the preferred use of GGU may originate from the stacking properties of C_32_ in tRNA_Gly_‐U_34_CC (Claesson et al., 1995; Lustig et al., 1993) or the combination with the second tRNA_Gly_ also reading GGU.

Finally, the NNU/C duet boxes show the largest difference in usage between low‐ and high‐expression groups (Figures 4 and 8). In these duet boxes, each tRNA contains a G_34_ in the anticodon that shows a pairing efficiency for NNC codons that is about three times higher than that to NNU codons (Chan et al., 2017; Grosjean & Westhof, 2016). Interestingly, highly expressed genes tend to overuse NNC codons despite the directional mutational bias to increase translation efficiency, while low‐expression genes succumb to mutational bias and rather use NNU codons (Figure 8). The concordance of tRNA pairing energy and codon usage bias for the NNU/C duet boxes is hidden when looking at total CDSs, but is revealed when the focus is on highly expressed genes.

In conclusion, the tRNA set of the chloroplast is fairly well adapted to the nucleotide composition equilibrium caused by the mutational bias. The effect of tRNA concentrations affects the codon usage of only a few amino acids and correlates with the mutational bias, while codon–anticodon pairing affinity appears as the dominant factor dictating codon usage and consequent bias in the chloroplast. The interactions between the anticodon loop and the mRNA strand are not exactly restricted to the anticodon itself, especially concerning interactions with ribosomes; therefore, a codon context dependency emerges that may influence codon choice. We will now explore if such a contextual effect is present in the chloroplast.

#### The influence of codon context on codon choice

Protein function and features dictate the amino acid composition of protein primary sequences; hence, the use of the appropriate codon family at each position of coding mRNA primary sequences is imposed. As presented above, the choice among redundant codons for an amino acid is determined based on codon usage bias. In addition, different types of protein features (such as α‐helices) present diverse composition enrichments in certain categories of amino acids (e.g., aliphatic amino acids) due to their functionality and substitutability (Grantham, 1974; Yampolsky & Stoltzfus, 2005). The non‐randomness in the juxtaposition of amino acids constitutes the dipeptide bias (Ghadimi et al., 2018), i.e., two amino acids are associated with a different frequency than their coupled respective frequency of occurrence.

Besides codon usage and dipeptide biases, another mechanism related to codon context was hypothesized to influence codon choice in coding mRNAs. Across all three kingdoms of life, the existence of a species‐specific codon pair bias was evidenced (Buchan et al., 2006; Moura et al., 2007, 2011; Tats et al., 2008). There are fundamental differences in the mechanisms for this codon bias between eukaryotes and prokaryotes. In eukaryotes, codon pair bias was shown to be a direct consequence of dinucleotide bias and DNA methylation at the junction of two codons (Kunec & Osterrieder, 2016; Moura et al., 2007). In prokaryotes, however, codon pair bias seems to arise from constraints imposed by the translational machinery (Boycheva et al., 2003; Moura et al., 2007, 2011). While there are no common preferred codon pairs across organisms (Moura et al., 2007), each bacteria seems to specifically overuse certain codon pairs in relation to their respective translation apparatus (Buchan et al., 2006; Moura et al., 2007; Moura et al., 2011).

In order to identify a potential influence of codon pair bias in the chloroplast, we sought to compare the actual occurrence of codon pairs in CDSs with their expected occurrence based on codon usage of individual codons. We first removed the effect of dipeptide bias to match over/underrepresented dipeptide associations (Buchan et al., 2006; Ghadimi et al., 2018) (see Methods and Data S4). After this step, we compared the number of codon pairs observed in CDSs with expectations calculated from a random codon distribution for the 3721 possible combinations (61 × 61 codons) (see Methods and Data S4). Caution must be applied with the calculation of the codon pair bias because the method relies on a probabilistic model using codon counts and codon frequencies. Therefore, this method is not suitable for the prediction of rare events, as is the case for pairs involving rare codons. As an illustration, the rare leucine codon CTC, which is present only 15 times in the genome, will show by default a low probability of pairing with any other codons (below one occurrence or very low frequency). This low expected occurrence/frequency will artificially inflate the codon pair bias as soon as a pair is present because of the division by an artificially low number. To avoid this issue, some studies excluded rare codons from the analysis (Buchan et al., 2006; Gutman & Hatfield, 1989); others did not take it into consideration, leading to artificial inflation of codon pair bias by including rare codons. Since rare codons constitute a significant fraction of sense codons in the chloroplast, we aimed to include them in the analysis. To circumvent distortions by low‐frequency codons, while preserving rare codons in the analysis, we adopted a mixed approach. This approach consists of a conventional probabilistic model for common codons and a deterministic model for rare codons (see Methods). In brief, a Poisson probability law was used to assess the potential of each codon to make on average one pair or less with a 75% confidence threshold. The codons falling into that category were considered to be rare and therefore followed a deterministic model. For simplification, both models follow the same calculations for the number of expected pairs, but the value is rounded up to the nearest whole number to avoid artificial inflation of the codon pair bias. We present the differences between our analysis based on these two models and an analysis that does not consider the issue of artificial inflation in the calculations (Figure S1).

The analysis of codon pair bias indicates that there are far fewer overrepresented codon pairs than suggested by previous studies. Although there are still some overrepresented codon pairs in the chloroplast, it is difficult to draw conclusions from the candidates showing deviations from expectations. It is important to note that the relatively low total number of codons in the chloroplast may hide some existing traits of codon pair bias.

**Figure 9:**
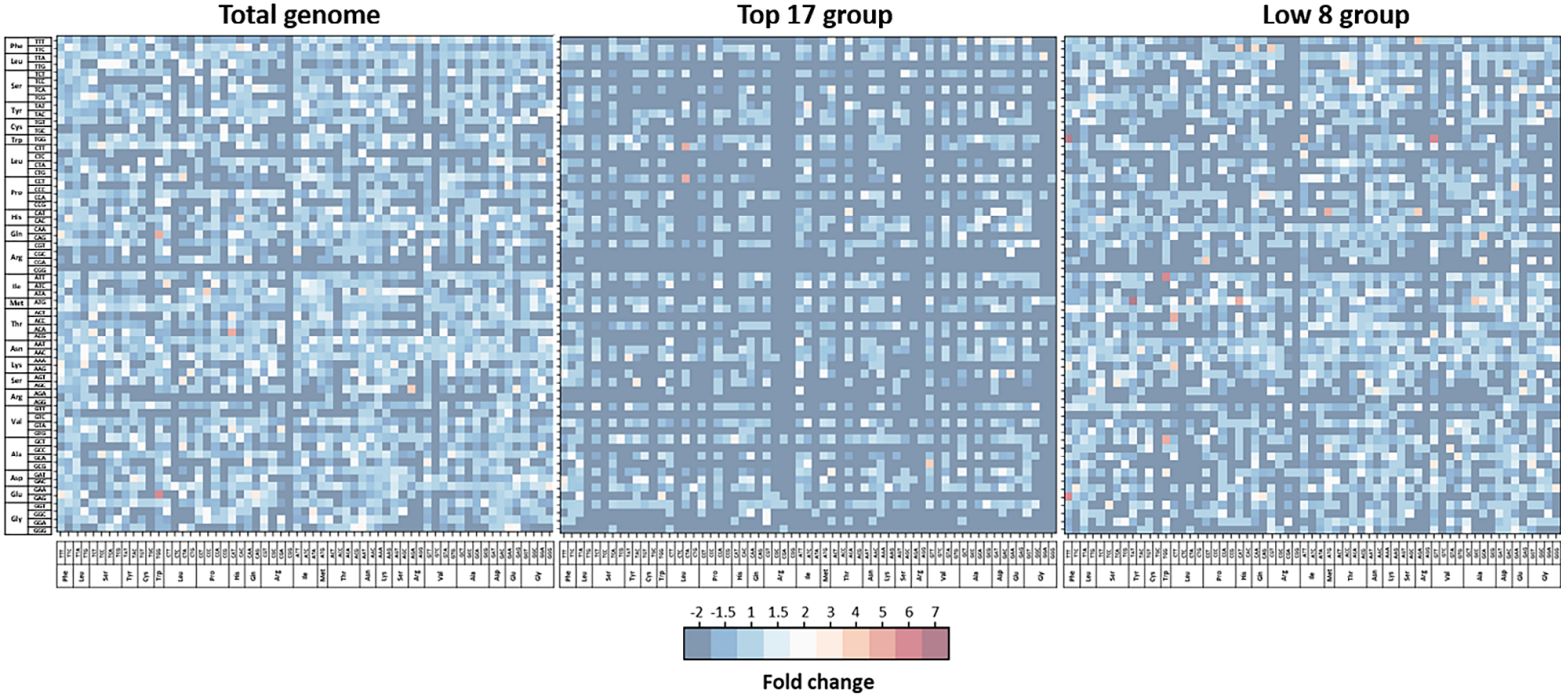
Codon pair bias of different expression groups. The colored matrix represents codon pair bias within each expression group. Blue indicates underrepresented codon pairs, white indicates the absence of significant bias, and red indicates significantly overrepresented codon pairs (>3‐fold). Each codon with their encoded amino acids is represented on the *y*‐axis (codon 1: ribosomal P‐site) and *x*‐axis (codon 2: ribosomal A‐site). Since rare events were taken into consideration for the calculations, there are no artificially overexpressed codon pairs. Thus, the number of significantly overrepresented codon pairs is relatively low, and their identity often differs between groups. These matrices may indicate the presence of codon context dependencies influencing codon choice. Data are available in Data S4.

The results presented in Figure 9 show under/overused codon pairs in the genome and across expression groups. Since underused codon pairs are very common due to the deterministic model and the low usage of certain codons, we focused solely on overused pairs. Indeed, as mentioned above, bacteria tend to overuse certain codon pairs (Buchan et al., 2006); we have extracted several codon pairs from chloroplastic CDSs overrepresented more than 3‐fold compared to expectations (Data S4).

A first observation is that most overrepresented codon pairs contain at least one translationally unfavorable codon, as exemplified by the following pairs: Arg‐Arg (AGA‐AGA), Ile‐Pro (ATA‐CCC), Ala‐Val (GCG‐GTT), Glu‐Gln (GAG‐CAG), and Leu‐Thr (TTG‐ACC). Codon pair bias can result from the juxtaposition of unfavorable codons acting in concert to slow down translation (Irwin et al., 1995). Indeed, the occurrence of a codon pair bias was correlated with translation efficiency and accuracy (Gamble et al., 2016; Kurland, 1987; Shpaer, 1986) and with the presence of protein tertiary structures (Widmann et al., 2008).

Although mechanisms involved have not been fully identified, the tRNA wobble base interaction with the third nucleotide of the P‐site codon (cP_3_) and at least the first, up to all three nucleotides of the A‐site codon (cA_1,2,3_), have been correlated with codon pair bias (Bossi & Roth, 1980; Buchan et al., 2006). In the chloroplast, codon context preferences were observed with the first nucleotide of the adjacent codon (cA_1_) (Morton, 2003). In addition, it was hypothesized that tRNA–tRNA interactions in the ribosomal A‐ and P‐sites may influence translation efficiency, thus shaping codon pair usage (Buchan et al., 2006; Smith & Yarus, 1989). In the case of the chloroplast, there seems to be no relationship between the types of aminoacyl‐tRNA either in the P‐ or A‐site and overused codon pairs. Since most codon families are decoded by only one tRNA species, it is unlikely that translationally detrimental tRNA–tRNA interactions were sustained throughout evolution. Although it could be the case for amino acids decoded by several tRNAs (Leu, Ser, Arg, Gly, and Ile), we found no evidence of preferences between their two respective tRNA isoacceptors for any codon family either in the A‐ or P‐site. The evolutionary tendency toward choosing a preferred tRNA isoacceptor for these amino acids may have started from the lack of detrimental tRNA–tRNA interactions before concentrations were optimized. In the chloroplast, it is difficult to draw conclusions from this analysis, due to the very low occurrence of certain codons, which drowns out a potential effect of tRNA–tRNA interaction.

Across all kingdoms of life, evolution suppressed NNU_3_‐A_1_NN and NNU_3_‐G_1_NN motifs in codon pairs to avoid potential out‐of‐frame UAA and UGA stop codons (cP_3_‐cA_1,2_) (Moura et al., 2007; Tats et al., 2008). In addition to stop codons, codon pair bias tends to avoid mononucleotide repeats that can lead to frameshifts and loss of protein function (Berg & Silva, 1997; Gu et al., 2010; Tats et al., 2008). In the chloroplast, we found no particular repression involving overlapping stop codons either in cP_3_‐cA_1,2_ or cP_2,3_‐cA_1_. Similarly, we found no active avoidance of tetra‐, penta‐, or hexanucleotide repeats (Data S4).

Dinucleotide bias at the junction of two adjacent codons is responsible for codon pair bias in eukaryotes, but it is not evident in bacteria (Kunec & Osterrieder, 2016; Moura et al., 2007). We analyzed the proportion of the 16 potential overlapping dinucleotides in all overrepresented pairs. We found a slightly higher proportion of dinucleotides corresponding to G or C in the third position of the P‐site codon (cP_3_); AT and GC contents of the cP_3_ position are 41 and 59%, respectively (Data S4). However, the higher presence of GC in cP_3_ is simply a consequence of the overuse of translationally unfavorable codons, usually containing G or C in the third codon position of chloroplastic CDSs.

A correlation between overuse of certain codon pairs and gene expression was initially hypothesized (Gutman & Hatfield, 1989; Yarus & Folley, 1985), but later questioned due to the low number of genes in the initial analysis. Correlation of codon pairing with gene expression was demonstrated to be only moderate (Boycheva et al., 2003; Buchan et al., 2006). We found that there are only a few overrepresented pairs in the Top expression group and a larger proportion in the Low expression group. Although some of these pairs are present across groups, such as the Ala‐Val pair GCG‐GTT, most overrepresented pairs differ among the three expression groups (Figure 9 and Data S4). Thus, there is no correlation between gene expression and the identity of particular codon pairs. However, there is an increase in the total number of overrepresented pairs with relatively low expression.

To conclude, codon pairing does not seem to be a major mechanism for shaping codon usage in mRNA CDSs that can be separated from codon usage bias. The effects of codon context might not be preponderant in the chloroplast due to its minimalistic nature or might be difficult to detect due to the very low occurrence of rare codons.

### Functional implications of codon usage bias in the chloroplast context

In this final section, we show the impact of codon usage bias on cellular processes leading to protein expression. In addition to optimizing protein translation rates, codon usage bias and amino acid usage may also provide relative pauses for protein folding and affect mRNA secondary structures involved in ribosome recruitment. Furthermore, it has been hypothesized that clusters of rare codons decrease early translation rates due to space limitation of ribosomes along the mRNA strand.

#### Codon usage affects translation rates and protein yield

The main purpose of codon usage bias is to optimize translation yield and accuracy and conserve protein structure and functionality, while managing resources to optimize cell fitness. The average translation rate in prokaryotes is about 20 amino acids per second (Gouy & Grantham, 1980) and can vary by more than one order of magnitude (Chevance et al., 2014; Sørensen et al., 1989; Sørensen & Pedersen, 1991). Ribosome profiling studies aim to identify factors influencing translation speed; however, limits in sensitivity allow to focus mainly on the most extreme translational pauses, for example those caused by internal SD sequences (Li et al., 2012). New analysis designed to quantify comparatively subtle differences in translation rate demonstrated that rare codons are translated more slowly than synonymous optimal codons (Gardin et al., 2014; Stadler & Fire, 2011). Indeed, all the factors contributing to codon usage bias were demonstrated to affect the translation rate. The additive effect of decreased translation speed originating from rare codons constitutes a key regulatory factor affecting low‐expression genes. In contrast, translationally optimal codon usage increases protein production of highly expressed genes (as shown in Figure 2). Both factors responsible for codon usage bias, tRNA concentrations and codon–anticodon pairing affinity, influence translation rates and protein yield.

##### The biological implications of tRNA concentrations

A predominant factor that determines the efficiency of translation is the availability, turnover, and relative concentrations of tRNA isoacceptor species (Gouy & Grantham, 1980; Pedersen, 1984; Varenne et al., 1984). The entry events of rare tRNA isoacceptors into the ribosome A‐site are less frequent, making the ribosome stall while waiting for the correct tRNA isoacceptor, thus slowing down translation. In the chloroplast, only a handful of amino acids are decoded by several tRNAs (Leu, Arg, Ser, Ile, and Gly). Since highly expressed genes are biased toward an increased translation yield, we correlated codon usage for these amino acids with their respective tRNA isoacceptor concentrations in Section 2.2 (Figure 3 and Figure 4). Briefly, leucine uses preferentially the UUA/G duet box for optimized translation; arginine and serine use rather their quartet boxes CGN and UCN, respectively; isoleucine uses the rare AUA codon to regulate translation of low‐expression genes, while AUU is the translationally favored codon; glycine might use its two tRNA isoacceptors to favor the GGU codon, although here, tRNA concentrations are not the most significant parameter. The tRNA concentration effects can be assessed by examining the correlation between codon usage bias and tRNA properties presented in Figure 8. The reliance on codon–anticodon pairing energy inside a codon box is decreased because the second box offers alternative codons. For example, the serine codon AGC of the duet box would be expected to be used preferentially in highly expressed genes; instead, the optimal UCU/A codons from the quartet box are used (Figure 8). Therefore, codon usage bias of the serine duet box is not correlated with the pairing energy of tRNA_Ser_‐G_34_CU. Interestingly, for these amino acids, tRNA concentration‐mediated codon regulation concords with a directional mutational bias toward AT_3_, so the pressure to counterbalance mutational bias toward C‐ending codons is less severe (as presented in Section 2.1). It is interesting to note that regulation of aminoacyl‐tRNA levels, charging, and modifications can cause changes in the set of genes preferentially expressed, for example due to the cell cycle, growth conditions, or circadian rhythms (Jayabaskaran et al., 1990; Matsuo et al., 2006; Nedialkova & Leidel, 2015).

##### The effects of codon–anticodon pairing affinity on translation rates

Another dominant factor affecting translation is codon–anticodon pairing affinity (Grosjean & Westhof, 2016; Letzring et al., 2010). Indeed, perfectly matching Watson–Crick base pairing between codon and anticodon decreases the time necessary to recruit and utilize the aminoacyl‐tRNA in comparison to wobble base pairing at the third codon nucleotide (Grosjean & Westhof, 2016; Letzring et al., 2010; Stadler & Fire, 2011). Therefore, translation efficiency increases with codon–anticodon pairing stability and interaction with ribosome geometry (Grosjean & Westhof, 2016; Letzring et al., 2010).

We previously presented the codon–anticodon affinities for the three groups of codons in Section 2.3. Briefly, the NNA/G duet boxes strongly favor A‐ending codons (Grosjean et al., 2010; Kurata et al., 2008; Lim, 1994; Takai & Yokoyama, 2003), which benefits translation rates, while overuse of NNG codons could be detrimental to protein expression yield. In quartet boxes, the modified or unmodified U_34_ of anticodons generally shows a strong preference for U‐ and A‐ending codons (Grosjean & Westhof, 2016) (for exceptions see Section 2.3), which should be reflected in high translation rates for these codons. The NNU/C duet boxes present an affinity for NNC codons around three times higher than for NNU codons. Here, both codons can be distributed along a CDS to modulate translation yield without severe consequences.

In order to verify the effects of codon–anticodon pairing affinity on translation rates, we examined previous data that analyzed ribosomal proteogenic site occupancy by aminoacyl‐tRNAs (Gawroński et al., 2018). Interaction of tRNA with mRNA in ribosomes can be evaluated by ribosome profiling. This method analyzes ribosomal‐protected mRNA fragments to establish enzyme densities along coding mRNAs. From these data, pausing sites can be extracted based on the assumption that an increase in ribosomal density reflects a decrease in translation speed. In a previous study aiming to identify the major translational pauses in the chloroplast of *Arabidopsis thaliana*, Gawroński et al. did not find a correlation between codon usage and ribosomal pausing (Gawroński et al., 2018). However, this analysis was not flawless, because it used the genomic codon usage, which often causes erroneous conclusions regarding codon usage bias. We performed a new analysis of their data, taking into consideration that translationally optimal codons are reflected in high‐expression genes due to the arguments presented in previous sections. While the sensitivity of the method may not provide the required accuracy for precise quantification of codon translation speed, it is possible to extract tendencies. We analyzed the relative pausing score of allegedly favored over detrimental codons for all codon boxes when they are present in the P‐site only, EP‐sites, and EPA‐sites. Overall, apparent translation speed correlates with tRNA pairing affinity and codon usage as presented in Figure 8 with the exception of threonine, glutamate, and aspartate. For NNA/G duet boxes, the G‐ending codons present an average pausing score that is 49% higher than their A‐ending counterparts (69% excluding glutamate). For NNC/U duet boxes, the G_34_:U_3_ wobble base pauses 68% longer than the G_34_:C_3_ Watson–Crick pair (80% excluding aspartate; 38 and 48% when considering the EP‐sites, respectively). Quartet boxes show an on average 35% increased pausing score for unfavorable codons versus their optimal counterparts (47% excluding threonine, 38 and 50% in the EP‐sites, respectively) (Data S5). These results are in accordance with the finding that codons deciphered through Watson–Crick codon–anticodon pairing are translated faster than their synonymous wobble‐pairing codons (Wang et al., 2017). In addition, it was shown that optimal and frequent codons were decoded more quickly than rare codons and that AT‐rich codons were translated faster than GC‐rich codons (Gardin et al., 2014). We can also distinguish a longer decoding time for tRNA with hypothesized lower concentrations than the second isoacceptor (for Ile, Leu, and Arg), but not for serine since similar tRNA concentrations were postulated. These data point toward our model of codon optimality in chloroplasts. Nonetheless, it is important to note that *C. reinhardtii* and *A. thaliana* are relatively distant species and may present some evolutionary differences regarding this topic. Although these relative changes in translation kinetics are subtle, their additive effects constitute a major factor that affects translation yield. We mapped all codons considered as translationally ‘slower’ along all CDSs (Figure S2), and it is clear that there is a gradient from rarely present toward abundant with decreasing gene expression.

#### The amino acid composition influences translation rates

**Figure 10:**
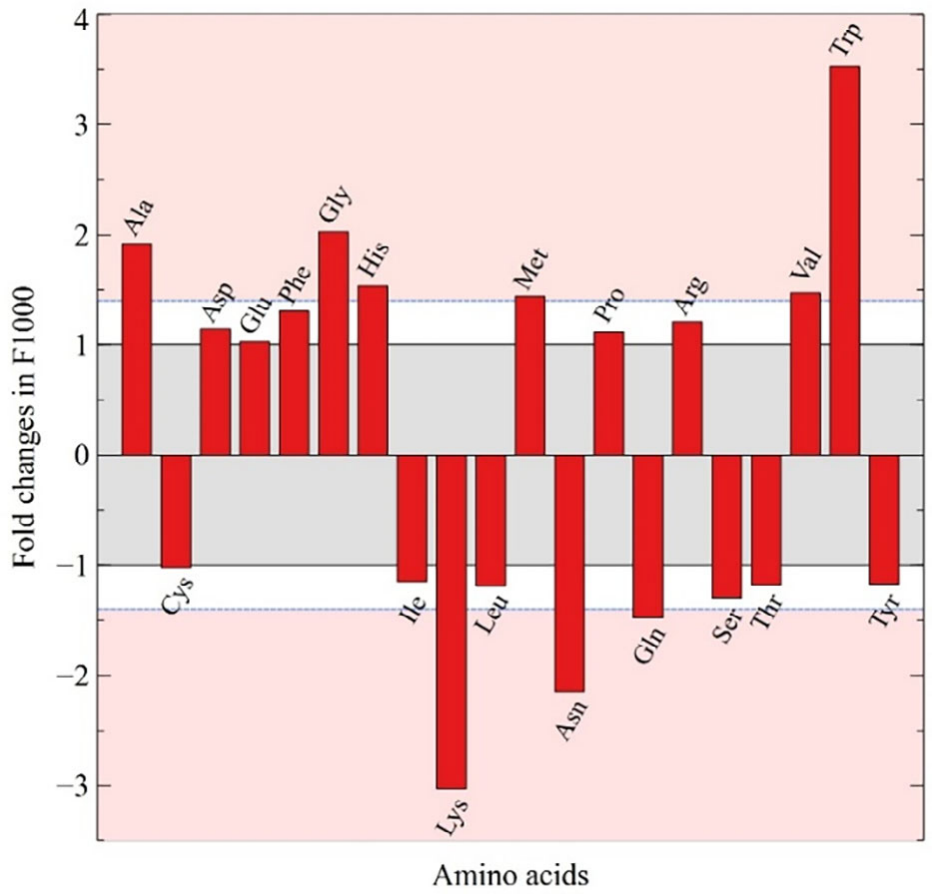
Fold changes in amino acid composition. The changes in amino acid composition of proteins (frequency per thousand [F/1000]) between the Top 18 and Low 8 expression groups are shown. Red areas represent fold change values considered significant (>1.4‐fold).

Similar to the availability of tRNA, regulation could also be exerted through a differential usage of amino acids in proteins from distinct expression groups; however, analysis of the amino acid bias did not show the presence of such a mechanism in the chloroplast. As described before, there is a directional nucleotide mutational bias resulting from an equilibrium in transversion kinetics (Sueoka, 1962; Sueoka, 1988). However, the effect of directional mutational bias is not restricted to neutral regions and strongly influences the amino acid composition of proteins (Gu et al., 1998; Knight et al., 2001; Lobry, 1997; Singer & Hickey, 2000; Sueoka, 1961). Evidently, the determining factor of amino acid composition results from selection based on their functionality (Lobry & Gautier, 1994). For example, membrane proteins are enriched in hydrophobic amino acids while cytoplasmic proteins contain more hydrophilic amino acids. The amino acid bias correlates with GC content and is exerted in near‐neutral sites of proteins (Osawa et al., 1990, 1992) or through similar amino acid characteristics (Grantham, 1974; Yampolsky & Stoltzfus, 2005). Hence, higher GC content correlates with an increase in codons containing one or two G:C bases while higher AT content favors codons with two or three A:T bases. The difference in GC content between high‐ and low‐expression genes (39.0 and 30.9%, respectively) is responsible for their bias in amino acid composition. The high‐expression set is clearly enriched in Ala, Gly, Trp, His, Val, and Met (40% increase) and to a lower extent in Pro, Arg, Asp, Glu, and Phe (Figure 10). This is in contrast to the low‐expression set, which is enriched mainly in Lys, Asn, and Gln (40% decrease) and to a lower extent in Tyr, Ile, Leu, Thr, and Ser (Figure 10). Last, it is interesting to note that the positively charged amino acid lysine is used 3‐fold more in low‐expression genes. The peptide exit tunnel of the ribosome is composed of negatively charged residues and it has been hypothesized that interaction with peptides enriched in positively charged amino acids slows down translation (Charneski & Hurst, 2013; Lu & Deutsch, 2008).

#### The impact of codon usage on protein folding

In addition to encoding the primary sequence of a protein, mRNA possesses additional functions. While optimal codon usage and amino acid composition improve protein yield, an overabundance of optimal codons increases the amount of insoluble or misfolded proteins, illustrating the necessity for rare codons (Angov, 2011; Crombie et al., 1992; Jacobson & Clark, 2016; Komar et al., 1999; Rosano & Ceccarelli, 2009; Siller et al., 2010; Spencer et al., 2012). Indeed, fine‐tuned translation rates ensure protein quality and cotranslational folding efficiency by inducing ribosomal pausing around protein domain boundaries, leading to the proper folding pathway (Buhr et al., 2016; Kaiser et al., 2011; Purvis et al., 1987; Thanaraj & Argos, 1996; Tsai et al., 2008). In bacteria, a local decrease in translation speed affects cotranslational protein folding, cofactor binding, multi‐domain assembly, chemical modifications, and membrane targeting (Gloge et al., 2014). In the chloroplast, in addition to accurate protein folding, a multitude of cofactors, such as pigments, quinones, hemes, and metal ions, are cotranslationally associated with nascent proteins (Nickelsen & Rengstl, 2013; Schöttler et al., 2011, 2015). Specific ribosomal pausing sites along coding mRNA, for *psbA*, *psaA*, *psaB*, and *psaC*, were suggested to facilitate the integration of stabilizing cofactors (e.g., chlorophyll) and promote the accurate folding and biogenesis of photosystem I and II multi‐protein complexes (Gawroński et al., 2018; Kim et al., 1991, 1994; van Wijk et al., 1996). Another important factor for the chloroplast is the targeting of nascent proteins to the thylakoid membrane, which occurs cotranslationally for a significant fraction of plastidial proteins (Celedon & Cline, 2013; Friemann & Hachtel, 1988; Margulies et al., 1987; Zoschke & Barkan, 2015).

The various mechanisms responsible for codon usage that affect translation kinetics were correlated with enhanced cotranslational protein folding (Komar, 2009; Zhang & Ignatova, 2011). Altering these parameters through synonymous codon substitutions lead to slight differences but substantial consequences on protein structure, function, and cell fitness (Buhr et al., 2016; Tsai et al., 2008; Walsh et al., 2020; Zhang et al., 2009). The ribosomal exit tunnel can accommodate 30–40 amino acids of the nascent peptide chain and allows the formation of secondary structures such as α‐helices (Bhushan et al., 2010; Gloge et al., 2014; Holtkamp et al., 2015). However, tertiary structures form when polypeptides exit the tunnel (Holtkamp et al., 2015; Lin et al., 2012; Lu & Deutsch, 2005); thus, there is a polypeptide spacer between the pause‐causing event and the formation of the tertiary structure. The pausing events can be caused at the P‐site (e.g., related to codons/tRNAs), upstream (e.g., SD sequence or positively charged amino acids), or downstream from the P‐site (e.g., mRNA structure).

One of the major mechanisms causing strong ribosomal pauses is mRNA secondary structures (Chen et al., 2013; Qu et al., 2011). The duration of ribosomal pauses depends on the strength of these structures and reflects the ability of the ribosome to remove these obstacles (Chursov et al., 2013; Qu et al., 2011). In the chloroplast, it was shown that pauses due to mRNA secondary structures are the main factor influencing protein domain folding (Gawroński et al., 2018). Codon choice can indirectly affect protein expression by influencing mRNA secondary structures, hindering or improving transit of ribosomes during translation (Chursov et al., 2013; Qu et al., 2011). Synonymous codon substitutions can disrupt mRNA secondary structures; therefore, they can affect the proper folding of protein domains. This may explain why a few rare codons remain in specific positions of high‐expression genes that can be conserved across species (Chursov et al., 2013; Pechmann & Frydman, 2013).

The second mechanism proposed to explain ribosomal pauses is the presence of an internal SD sequence that interacts with the anti‐SD sequence of the ribosomal 16S RNA (Li et al., 2012). However, some of these claims have been disputed and assigned to technical artifacts (Mohammad et al., 2016). In chloroplast of *C. reinhardtii*, the anti‐SD sequence is 3′‐UCCUCC‐5′, corresponding to a 5′‐AGGAGG‐3′ SD sequence. In plastidial CDSs, exactly matching penta‐ and hexanucleotide SD sequences rarely occur (a total of nine times in the Low 8 group). While tetranucleotides matching the SD sequence, such as AGGA, GGAG, or GAGG, are more common, they are rare in the high‐expression group. To consider imperfect matches, Gawroński et al. calculated the hybridization energy of an 8‐nucleotide window with the anti‐SD sequence of the chloroplast. Their results indicate that the strongest anti‐SD–mRNA interactions upstream of the pause sites correlate with the duration of the pausing event (Gawroński et al., 2018). Interactions with the anti‐SD sequence require a certain nucleotide composition, especially rich in guanosine. The corresponding codons potentially interact with the anti‐SD sequence and often represent translationally unfavorable codons, such as glycine GGA, glutamate GAG, and arginine AGG. As for mRNA secondary structures, these codons might be sustained in specific positions of coding mRNA in order to maintain anti‐SD‐related ribosomal pausing.

In addition to these two mechanisms, the presence of positively charged amino acids in the nascent polypeptide chain were shown to interact with the negatively charged ribosomal exit tunnel, thus causing a decrease in translation rate (Charneski & Hurst, 2013; Lu & Deutsch, 2008). In the chloroplast, the positively charged amino acids (lysine, arginine, and histidine) conferred ribosomal pauses, which were, however, weaker than those caused by the two previous mechanisms (Gawroński et al., 2018). We showed in the previous section that low‐yield proteins are enriched in lysine by a factor of 3 compared to high‐yield proteins. This distribution bears witness to the evolutionary pressure applied on the protein primary sequence that also shapes protein expression (Gu et al., 1998; Knight et al., 2001; Lobry, 1997; Singer & Hickey, 2000; Sueoka, 1961). The distribution of lysine residues along a protein's primary sequence induces small decreases of translational speed that may correlate with minor requirements of protein tertiary structures.

The three mechanisms influencing ribosomal pausing mentioned in the previous paragraphs can be directly affected by synonymous codon substitutions. Since recoding of synonymous codons was demonstrated to disrupt protein functionality and increase misfolding (Rosano & Ceccarelli, 2009; Widmann et al., 2008; Zhang et al., 2009), seemingly unfavorable codons might be sustained in specific positions to ensure protein quality. Across diverse eukaryotic, bacterial, and archaeal genomes, homologous CDSs show conserved rare codon clusters separating small protein structural motifs (Chaney et al., 2017; Clarke IV & Clark, 2008). The positive selection for these position‐specific clusters, observed independently of gene expression, suggest a functional role in protein maturation (Chaney et al., 2017; Clarke IV & Clark, 2008). While individual rare codons decrease translation rates, it is not sufficient to induce ribosomal pausing that is sufficient for protein domain folding. These small pauses might be involved in less demanding structural properties (Jacobs & Shakhnovich, 2017). It is unclear if the cumulative effects of decreased translation rates from rare codon clusters can provide pauses that are long enough or if they act indirectly through the mechanisms presented previously. The occurrence of several consecutive rare codons might take advantage of low tRNA concentrations (Fedyunin et al., 2012; Parmley & Huynen, 2009), tRNA turnover and diffusion (Gouy & Grantham, 1980), and tRNA wobble properties to create an aggregate of reduction in translation rate (Zhang et al., 2009). On the contrary, some clusters contain repeats of the same rare codon to optimize translation, because the unusual proximity reduces the time necessary for tRNA turnover and diffusion (Cannarozzi et al., 2010). Nevertheless, these rare codon clusters were demonstrated to play a regulatory role in the folding of important domains (Chartier et al., 2012; Liu, 2020; Widmann et al., 2008; Zhang et al., 2009).

We mapped all the codons considered as translationally unfavorable on the CDSs of the chloroplast (Figure S2). First, we note that there is an increasing proportion of rare codons with decreasing gene expression, pointing toward the relationship between codon usage bias and protein yield. While some genes present a uniform distribution of their rare codons along the CDS, such as *rbcL*, other genes, like *atpA* and *psaB*, present small clusters of at least three unfavorable codons within a 30‐codon window. This accumulation is even more visible on intermediate‐expression genes such as *rps2* or *rps3*. For low‐expression genes, local increases in the density of rare codons are still discernable but are concealed by the relatively high general presence of these codons. Interestingly, some enrichments of rare codon occur in the 5′‐ or 3′‐termini of CDSs. This 5′‐ and 3′‐terminal enrichment has been linked to membrane targeting or protein secretion and to translation termination, respectively (Clarke & Clark, 2010; Gerresheim et al., 2020). In the chloroplast, significant enrichment of the 5′‐terminus with rare codons occurs for example in *psaA*, *psaB*, *psbB*, *rps3*, or *ycf1*, and this occurs at the 3′‐terminus in *rps4* or *rps7*.

In addition to the aforementioned mechanisms, codon context was demonstrated to affect translation kinetics (Chevance et al., 2014) but only moderately correlated with gene expression (Boycheva et al., 2003; Buchan et al., 2006; Chevance et al., 2014). Thus, the presence of overrepresented, unfavorable codon pairs was hypothesized to locally decrease translation rates in relation with protein maturation (Seligmann & Warthi, 2017). As presented previously, for high‐expression genes, there are only a few significantly overrepresented codon pairs and their total occurrence is relatively low. Moreover, since mechanisms responsible for codon pairing are not understood, it is difficult to estimate the contribution of codon context to protein maturation. Most overrepresented codon pairs possess at least one slow codon and are supposedly exploiting the first adjacent nucleotide (cA_1_) to intensify their kinetic effects on the ribosome. Thus, codon context may participate in protein maturation to a similar extent as, or in coordination with, rare codon clusters.

#### The relationship between mRNA structural features and codon choice influences protein expression

Protein expression yield depends on efficient translation initiation. The recruitment of ribosomes at the 5′‐untranslated region (UTR) of mRNA sequences is a crucial step for initiating translation. The recruitment process occurs either through interactions between the 5′‐UTR SD sequence and the anti‐SD sequence of the 16S rRNA or through non‐canonical translation initiation mechanisms (Chang et al., 2006; Nakagawa et al., 2017). In the chloroplast, although SD‐dependent recruitment certainly plays a role (Scharff et al., 2017), a large portion of genes use alternative mechanisms for ribosome recruitment (Fargo et al., 1998; Nakagawa et al., 2017; Scharff et al., 2011; Weiner et al., 2019). This type of translation initiation requires a low amount of mRNA secondary structure around the start of the gene. Indeed, efficient protein expression is a compromise between mRNA stability and ribosome entry site accessibility (Espah Borujeni et al., 2014, 2017; Espah Borujeni & Salis, 2016; Mignone et al., 2002). Plastidial SD‐less mRNAs show an increase in free energy from −30 to +20 nucleotides around the start codon, which is indicative of an absence of secondary structure (Scharff et al., 2011). The decrease in mRNA stability promotes ribosome recruitment and often correlates with a lower local GC content on each side of the start codon (Li & Qu, 2013).

Once ribosomes are recruited, translation initiation rates are influenced by processing of the start codon (Esposito, 2003) and the nucleotide composition at the start of the gene, a region referred to as the gene ‘ramp’ (T. Tuller, Carmi, et al., 2010a; T. Tuller, Waldman, et al., 2010b). The ramp is composed of the first 30 to 50 codons of CDSs and was shown to be enriched in slow codons (Fredrick & Ibba, 2010; T. Tuller, Carmi, et al., 2010a; T. Tuller, Waldman, et al., 2010b; Tuller & Zur, 2015; Villada et al., 2017). This enrichment in slow codons occurs across a wide range of organisms. These slow codons are not involved in protein cotranslational folding because the nascent polypeptide is still in the ribosomal exit tunnel. The presence of rare codon clusters at the 5′‐termini of genes was hypothesized to limit early translation rates, so as to allow spacing between ribosomes, hence avoiding traffic jams and ribosome fall‐offs (T. Tuller, Carmi, et al., 2010a; Zhang et al., 1994). However, it has been a point of debate whether the unusual codon usage in the ramp is solely a consequence of the selection for local mRNA secondary structures or these codons are present in order to slow down translation (Bentele et al., 2013; Goodman et al., 2013; Shah et al., 2013; T. Tuller, Carmi, et al., 2010a). Overall, the nucleotide composition of the ramp appears to be a compromise between its functional elements. The ramp starts with a decreased mRNA structure around the start codon, followed by a region enriched in slow codons and positively charged amino acids, which is followed by a stronger mRNA loop inside the ramp for stability (T. Tuller, Carmi, et al., 2010a; Tuller & Zur, 2015). In chloroplasts, it was demonstrated that not solely CAI dictates protein yield, suggesting that other parameters were equally important (Weiner et al., 2020).

To gain insight into the extent to which these different features are present in chloroplasts, we first quantified the actual enrichment of slow codons in gene ramps. We excluded from the analysis genes with a length of 150 nucleotides or below. We found that rare codons are 84% more frequent in the gene ramp compared to the rest of the mRNA sequence for the high‐expression group and 10% more frequent for the low‐expression group, but that there was no difference for intermediate genes (Data S6). Additionally, the occurrence of rare codons is highly gene‐dependent; some genes, such as *psbA* and *rbcL*, do not show any differential enrichment in rare codons for the ramp, while it is significant for other genes, such as *psaA*, *psaB*, *psbB*, *rps3*, and *ycf1*, which may indicate a special characteristic of membrane proteins (Figure S2). There is a slight increase in positively charged amino acids in the ramp of 25, 12, and 33% for low‐, intermediate‐, and high‐expression genes (Data S6). Furthermore, we analyzed the cumulative GC content of the first 30 codons as a proxy for secondary structure. There is an active pressure to decrease the GC content of the ramp up to the 10th codon for both high‐ and low‐expression groups (Figure S3) and before the start codon (Scharff et al., 2011). However, this pressure does not seem to act similarly on the nucleotide codon positions between the two expression groups. Finally, we also tested if recoding the rare codons of the ramps of *psaA*, *psaB*, and *psbB* with their favored synonymous counterpart had any effect on the mRNA structure of these genes (−50 to +150 nt). We found that recoding with common codons increased the minimal free energy of the centroid structure by 11.3, 3.8 and 1.7 kcal/mol for *psaA*, *psaB*, and *psbB*, respectively, and disrupted some mRNA structural characteristics. These results indicate that some rare codons within the ramp are involved in disrupting the mRNA secondary structures, either around the start of these genes or in the downstream stability loop. However, slow codons and charged amino acids may also be involved in limiting early translation rates.

## CONCLUSION

Our work advances our understanding of the complex interactions of codon usage with protein expression. First, we showed that codon usage is highly biased in correlation with the level of gene expression. Highly expressed genes have evolved to utilize a restricted set of codons that is deemed optimal. By comparing expression groups, we demonstrate that this codon optimality information is diluted by the genomic codon usage for proteins that require less optimal expression. This dilution effect was not identified by previous studies that investigated the relationship between codon usage and protein expression, thus leading to interpretations that lacked precision.

The optimization of highly expressed genes permitted to identify the favored codons, which obtain the highest protein yield. The directional mutational bias drives plastidial DNA composition toward an AT‐rich equilibrium. Directional mutational bias shapes codon usage of functionally less important genes, while for highly expressed genes other mechanisms counteract the mutational pressure to optimize their translation. In summary, optimal codon usage from duet boxes favors NNC over NNU codons and NNA over NNG codons. Quartet boxes favor their NNU/A codons, except for arginine and glycine, which favor only their respective U‐ending codons. While the usage of NNU/C duet boxes can be balanced to modulate expression, G‐ and C‐ending codons of NNA/G duet boxes and quartet boxes are actively avoided in highly expressed genes because their wobble base properties excessively affect translation rates. Additionally, tRNA concentrations affect codon optimality of leucine, serine, arginine, and isoleucine and determine which codon box is favored.

Codon optimality relates to ribosomal translation rates when encountering a given codon. Our analysis shows that tRNA characteristics such as anticodon loop modifications and codon–anticodon pairing affinity are the main factors determining translation rates. This is reflected in the codon usage optimality of highly expressed genes. Apart from protein yield, codon usage is involved directly or indirectly in protein cotranslational folding by influencing mRNA secondary structures, internal SD sequences, the codon context, and rare codon clusters. Synonymous substitutions in specific positions can persist through evolution, in order to maintain features causing ribosomal pauses. This is crucial for membrane targeting, protein domain folding, binding cofactors, such as chlorophyll or metal ions, or the integration of a protein to a multi‐enzymatic complex, such as photosystems I and II. Codon composition in the gene ramp is also modulated by mRNA structures and rare codon clusters, which regulate translation initiation.

**Figure 11:**
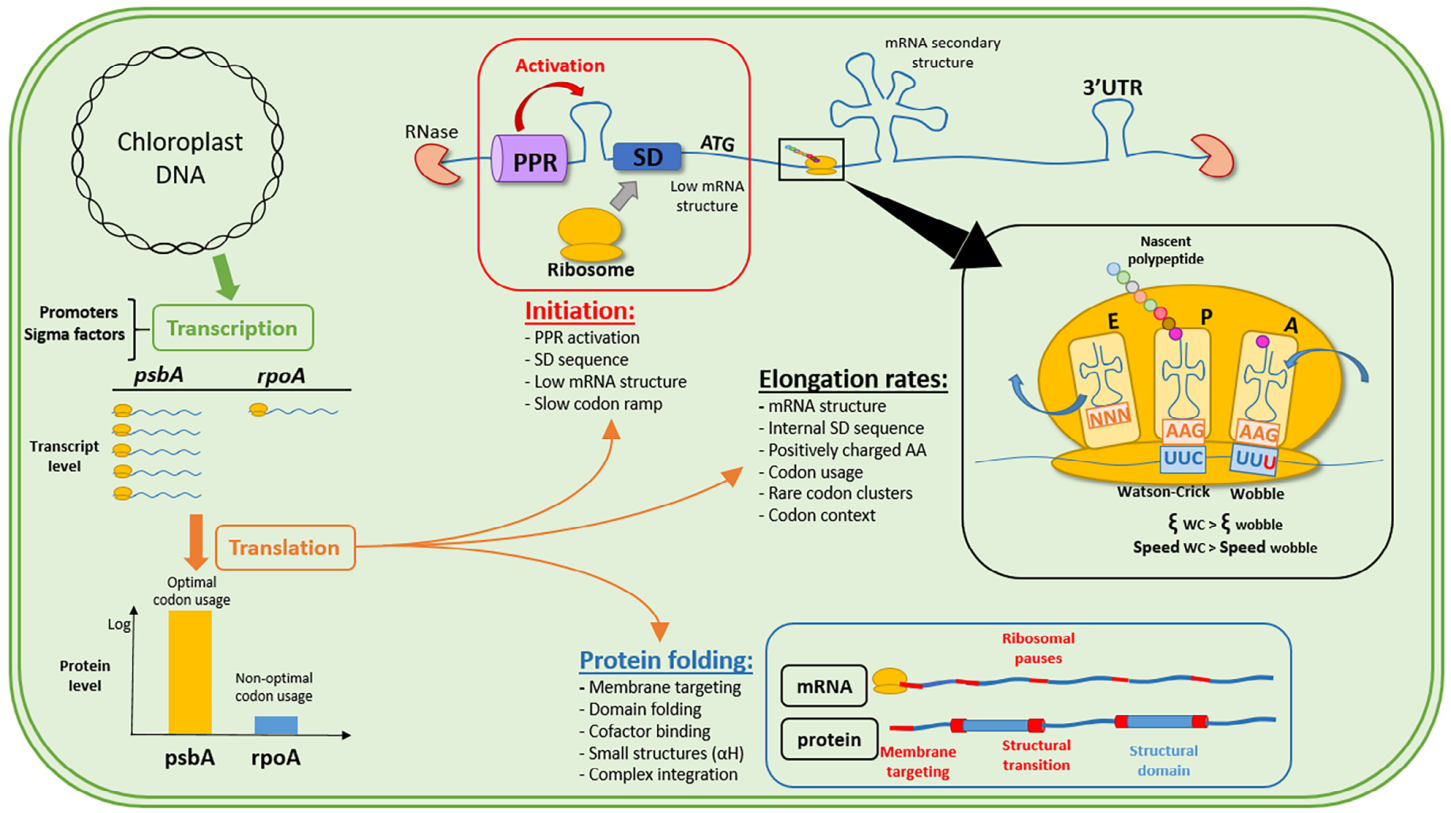
Summary of the main mechanisms affecting protein expression in chloroplasts. From plastidial DNA, genes are transcribed at different levels depending on promoter strength, sigma factors, and growth conditions. Translation yield is optimized through codon usage for high‐expression genes (represented by *psbA*), while codons from low‐expression genes are rather subject to mutational bias. At the transcript level, PPRs and mRNA structure stabilize the transcripts and enhance translation initiation (in addition to SD sequences). Enrichment in slow codons at the beginning of the gene (ramp) allows ribosome spacing to avoid traffic jams and fall‐offs. During elongation, codon usage and codon context modulate translation rates, while mRNA structures, internal SD sequences, and positively charged amino acids create longer ribosomal pauses. These pauses are necessary for protein cotranslational folding, cofactor binding, integration of proteins into multi‐protein complexes, or membrane targeting of proteins.

Overall, our results reconcile the codon usage of the chloroplast with eminent theories of bacterial codon usage (Figure 11). This includes codon usage bias associated with strong gene expression, the influence of mutational bias, and the codon–anticodon pairing affinity properties that modulate the deciphering ability of tRNAs and influence translation rates. Additionally, plastidial local codon usage is in line with the realization that mRNA contains more information than the primary amino acid sequence and is involved in translation initiation and the maintenance of protein features.

The comprehensive insights presented in this work will help codon optimization of heterologous genes for biotechnological applications in algae and plants. Additionally, the chloroplast appears as an entity of choice for advancing our understanding of the entanglement of genetic features with other cellular processes. The simplicity of the chloroplast provides unobstructed view at the first layers of the complexity of the genetic code, which cannot easily be gleaned from more complex organisms.

## METHODS

### Data

The *C. reinhardtii* chloroplast genome sequence CPv4 was retrieved from the supplementary information of Gallaher et al. (Gallaher et al., 2018) and annotated with the features of the previous version FJ423446.1 (Smith & Lee, 2009). Corrections in annotations were performed according to the work of Gallaher et al., especially for *rpoC1* and *rps2*, which are read as one single open reading frame (ORF). The mRNA quantification data of the chloroplast were retrieved from the same source (Gallaher et al., 2018). We used the mRNA sequencing data obtained under light conditions as an assumption for high/low gene expression, as transcriptomic data are much easier to obtain compared to quantitative proteomics and data are more accurate. Previous research establishing the principles for codon usage bias showed the correlation between codon usage, mRNA content, and protein content (Bennetzen & Hall, 1982; Gouy & Gautier, 1982, Ikemura, 1981; and for protein synthesis rates specifically for chloroplasts: Pfitzinger et al., 1987). These principles are the basis of codon optimization, where codon occurrence is correlated with tRNA content and protein synthesis rates. This previous research showed that transcripts could be used as proxy for the determination of codon usage bias under the assumption that translation can be associated to a proportional increase in the quantities of the encoded proteins. Two sets of genes were created based on high or low mRNA expression. The high‐expression set was composed of 18 genes (psbA*2, *rbcL*, *psaA‐B‐C*, *psbB‐C‐D‐F*, *atpA‐B‐H*, *ycf12*, *petB‐D*, *tufA*, *rps12*), and the low‐expression set contained 8 genes (*rpoA‐B1‐B2‐C1‐C2*, *chlB‐L‐N*) (referred to as Top 18 and Low 8, respectively) (Data S1). Non‐native plastid genes or not characterized genes such as I‐cre, Wendy transposons, or ORF 528 were excluded to avoid bias in codon usage analysis. At first, the reference set for the high‐expression group was composed of the seven genes (Top 7) that presented an FPKM value of >10 000 in RNAseq analysis (Data S1). The reference set was then extended to 18 genes (Top 18), with FPKM values of >5000, to include a higher number of codons, equivalent to the low‐expression group, in order to avoid biases. The two groups provide a very similar outcome in terms of correlation of codon usage bias (Data S2); hence, the largest group was kept for the analyses. The low‐expression group was composed of genes with FPKM values of <500, avoiding genes that could provide artifacts, due to lack of characterization or arising from potential RNAseq defects (e.g., *psbN* and *psbI*). The low‐ and high‐expression groups contain a similar number of total codons (8762 and 6151, respectively). Gene grouping did not affect much the correlation between CAI and expression, as the codon usage in the respective expression range is extremely close (Figure 2).

### Bioinformatics analysis

The number of codon occurrences in the total CDSs and the different expression groups was calculated with online resources (www.bioinformatics.org/sms2/) and a python‐based program we created (github.com/hundvin93/recodon‐python). Frequencies of occurrence per 1000 codons were used as normalized values. Codon usage tables for total CDSs and high‐expression gene sets were constructed from the codon usage database with the *countcodons* program (www.kazusa.or.jp/codon/countcodon.html) (Data S2).

### Codon usage calculations

We analyzed the RSCU, which is a measure of codon frequency, assuming equal codon usage for one amino acid. From the RSCU values, we calculated the relative adaptiveness of a codon W_ij_, which normalizes the codon frequency to the most used synonymous codon. This permits to identify the so‐called ‘optimal’ and ‘non‐optimal’ codons as frequent and rare, respectively. We used CAI (Sharp & Li, 1987) to assess codon usage bias. CAI uses a set of highly expressed genes hypothesized to possess the most optimal codons as a reference. The high‐expression gene sets (Top 7 and Top 18) were used as reference set for codon optimality. CAI values were computed using the code of Fox and Erill (Fox & Erill, 2010) (erilllab.umbc.edu/research/software/201‐2/). These calculations were performed on total CDSs and the different expression groups. Fold differences between groups were calculated for each codon (Data S2) as follows:

RSCU_ij_ = $\frac{\mathrm{Xi},j}{\frac{1}{\mathrm{ni}}\;\sum_{j=1}^{\mathrm{ni}}\mathrm{Xi},j},$

where *X*_*ij*_ is the number of occurrences of the *j*th codon for the *i*th amino acid and *n*_*ij*_ is the number of alternative synonymous possibilities for the *i*th amino acid (one to six). Moreover,

W_ij_ = $\frac{\text{RSCUi},j}{\text{RSCUi},\max}$ = $\frac{\mathrm{Xi},j}{\mathrm{Xi},\max},$

where the values for RSCU and occurrence *X*_*ij*_ are compared for the *j*th codon and the most frequent codon (*max*) for the *i*th amino acid. CAI was calculated as follows:

CAI = $\left(\prod_{j=1}^{L}\mathrm{Wj}\right)$ ^1/L^,

where *W*_*j*_ is the relative adaptiveness for the *j*th codon of a gene of codon length L.

### Codon pair calculations

The codon_pair.py function of the previously mentioned python program was used to perform the calculations (github.com/hundvin93/recodon‐python). The program extracts the observed occurrence (*o*_*ij*_) of all 3721 possible codon pairs (61 × 61) within CDSs. It also calculates the expected number of occurrences of a codon pair (*e*_*ij*_) similarly to previously described calculations (Buchan et al., 2006; Gutman & Hatfield, 1989):

*e*_*ij*_ = $\frac{C_{i}\;x\;C_{j}}{N_{p}}=c_{i}\times F_{j},$

where *c* is the number of occurrences of the *i*th and *j*th codons and *N*_*P*_ is the number of codon pairs (number of codons [*N*_*TOT*_] − 1). Using this relationship, for a fixed codon *i*, the probability of encountering a following codon *j* can be approximated to its frequency of occurrence (*F*_*j*_ *= c*_*j*_ */ N*_*TOT*_).

The expected codon pair frequency (*e*_*ij*_) was corrected for the dipeptide bias as previously described (Buchan et al., 2006; Gutman & Hatfield, 1989). This correction compensates the uneven distribution of amino acids along protein primary structures caused by their structural and functional properties. In brief, the over/underrepresentation of a dipeptide is estimated by dividing the actual occurrence of each amino acid pair (*P*_*kl*_), for the *k*th and *l*th amino acids, by the expected dipeptide frequency (*Q*_*kl*_) calculated from individual amino acid frequencies:

*dipeptide bias* (*DiPB*) = $\frac{P_{\mathrm{kl}}}{Q_{\mathrm{kl}}}.$

The expected frequency of occurrence for a codon pair (*e*_*ij*_) was corrected by the dipeptide bias to render the following equation:

*e*_*ij, norm*_ = $c_{i}\times F_{j}\times {\text{DiPB}}_{\mathrm{kl}},$

where *c*_*i*_ is the codon count for the *i*th codon, *F*_*j*_ is the frequency of occurrence of the *j*th codon, and *DiPB* is the dipeptide bias for the *kl* dipeptide (encoded by the *ij* codon pair).

The codon pair bias was calculated by comparing the number of occurrences of the observed codon pair (*o*_*ij*_) with its normalized expected frequency (*e*_*ij, norm*_) for a given codon pair *ij*:

*codon pair bias* (*ij*) = $\frac{\left(o_{\mathrm{ij}}\right)-\left(e_{\mathrm{ij},\mathrm{norm}}\right)}{\left(e_{\mathrm{ij},\mathrm{norm}}\right)}.$

However, this calculation creates an artificial overestimation of codon pair bias for rare codons. Indeed, the expected occurrence/frequency of pairs containing rare codons is very low; thus, the division artificially inflates the codon pair bias (Moura et al., 2005, 2007). Nonetheless, because rare codons exist, they will axiomatically pair with a *j* codon. Therefore, pairing events involving rare codons are semi‐deterministic. Some studies chose to exclude rare codons to avoid this artifact; however, in the chloroplast, rare codons represent a significant fraction and should be considered in this analysis. To circumvent this artifact, we first chose to work with the number of occurrences of codon pairs instead of their frequencies to relate the values obtained with their biological meaning. Then, we classified common codons following a probabilistic model and rare codons following a deterministic model. We used a Poisson law to assess the probability of each codon *i* to form maximum one codon pair with each codon *j*. The probability *P* of the number of occurrences (*X*) of a pair to be zero or one follows the law

$$P\left(X\leq k\right)=\sum_{k=0}^{1}\frac{\lambda^{k}}{k!}.e^{-\lambda },$$

where *k* is the possible number of occurrences of the codon pair *ij* and λ is the predicted frequency of occurrence of the event. For each pair *ij*, we have λ = $c_{i}\times F_{j}$. The average probability for each codon *i* to form only zero or one pair with all other codons was calculated and codons above a threshold of *P*(*X* ≤ 1) = 0.75 were considered to follow a deterministic model. Because of the deterministic nature, the expected number of codon pair occurrences for rare codons cannot be below one. For simplicity, we combined the probabilistic and deterministic models for common and rare codons, respectively, under the same codon pair bias calculation, but we rounded up the expected pair values to the nearest whole number. This way, rare codons are not excluded from calculations and do not provoke an artificial bias, although it makes most pairs involving rare codons artificially underrepresented.

### Calculation of free energy of RNA structures

The free energy of RNA structures was calculated using the web‐based RNAfold implementation (http://rna.tbi.univie.ac.at/). In order to assess if codons considered as rare present in the ramp have a significant impact on mRNA structures, they were substituted with one of their common counterparts.

## AUTHOR CONTRIBUTIONS

MFL and MHM conceived the study and wrote the manuscript. MFL performed the literature review and data analysis. KH wrote the python scripts and analyzed codon usage features.

## CONFLICT OF INTEREST

The authors declare no conflict of interests.

## Supporting information


**Figure S1**. Method comparison of codon pair bias calculations.
**Figure S2**. Gene maps of translationally unfavorable codons within expression groups.
**Figure S3**. Cumulative GC contents of the ramps of Top 18 and Low 8 groups.

**Data S1**. CAI analysis.

**Data S2**. Codon usage analysis within gene groups.

**Data S3**. tRNA copy number.

**Data S4**. Codon pair bias.

**Data S5**. Ribosome profiling.

**Data S6**. Codon usage within gene ramps.

## Data Availability

The supplemental data are accessible online at https://onlinelibrary.wiley.com. Expression data for mRNA sequencing were retrieved from the online supplemental material of Gallaher et al., 2018. The python programs we created were deposited on GitHub (githubcom/hundvin93/recodon‐python) and all other bioinformatic tools utilized in the study are listed in the Methods section.
