# Supplementary material for: Mechanisms governing codon usage bias and the implications for protein expression in the chloroplast of Chlamydomonas reinhardtii
Source: Plant J. 2022 Oct 19;112(4):919–45. doi: 10.1111/tpj.15970 (PMC9828097; doi:10.1111/tpj.15970)
Supplement: Supplementary file 1 — Figure S1. Method comparison of codon pair bias calculations. Figure S2. Gene maps of translationally unfavorable codons within expression groups. Figure S3. Cumulative GC contents of the ramps of Top 18 and Low 8 groups. [file TPJ-112-919-s001.pdf]

Supplemental Tables and Figures

"Mechanisms governing codon usage bias and the implications for protein expression in the chloroplast"

2021

Fages-Lartaud et al.

Tables of Content:

Supplemental Figure S1. Method comparison of codon pair bias calculations for each expression groups……… *p2*

Supplemental Figure S2. Gene maps showing the position of translationally unfavorable codons for three expression groups (High, intermediate, low)…………………………………………………………………………….. *p3*

Supplemental Figure S3. Cumulative GC contents of the ramps of Top 18 and Low 8 genes…………… *p4*

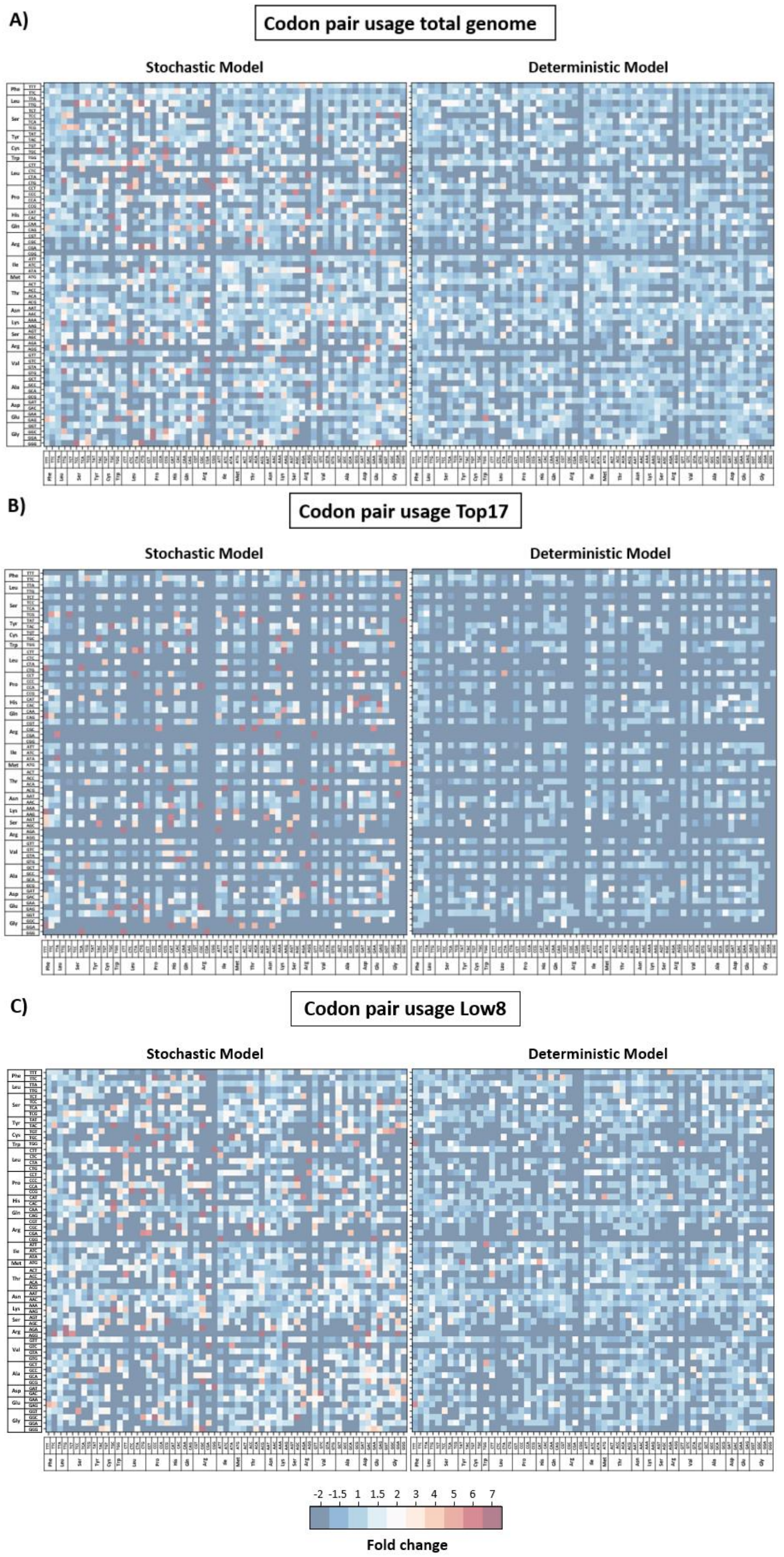

Supplemental Figure S1. Method comparison of codon pair bias calculations for each expression groups. The "stochastic" model refers to calculations used in the literature that artificially create a bias for rare codons. These calculations divide by an expected frequency (e_{ij}) that is by design very low, thus it inflates the difference with the observed codon pairs (Bias = (o_{ij} - e_{ij})/ e_{ij}). When considering rare codons under a deterministic model, we ensure that these codons will form either 0 or 1 pair. Under this assumption, we eliminate most artificial inflation of codon pair bias.

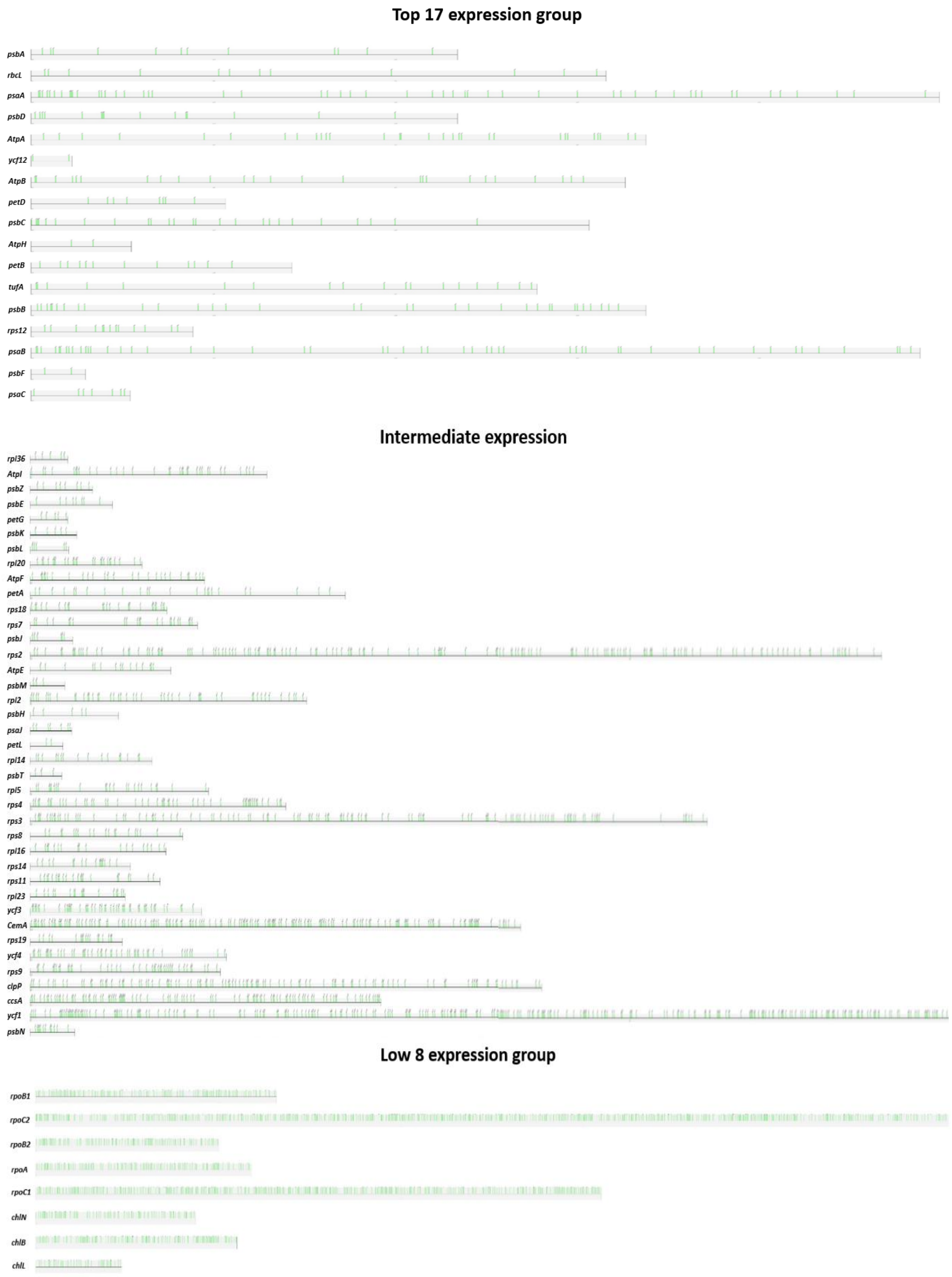

Supplemental Figure S2. Gene maps showing the position of translationally unfavorable codons for three expression groups (High, intermediate, low). The unfavorable codons represented here are listed in *Supplemental Data S1*.

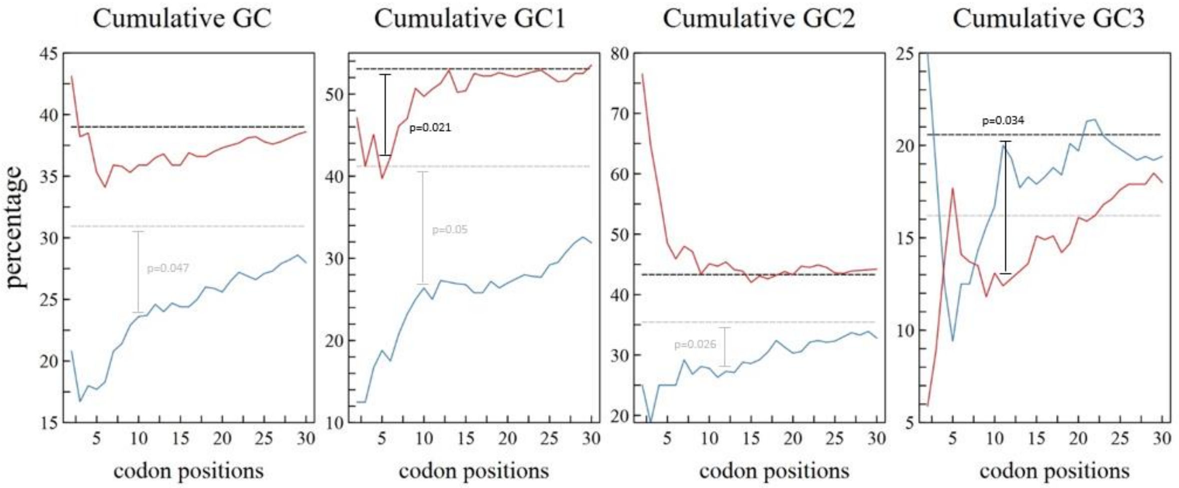

Supplemental Figure S3. Cumulative GC contents of the ramps of Top 18 (red) and Low 8 (blue) genes. The cumulative GC content of each codon position was calculated (GC1,2,3) and the total cumulative GC. The average for each value are represented with a dotted line, black for top 18 and grey for Low 8. Significant deviations from the average are shown with their respective p-values.
